# Artificial Intelligence in Intensive Care: An Overview of Systematic Reviews with Clinical Maturity and Readiness Mapping

**DOI:** 10.3390/jcm15010185

**Published:** 2025-12-26

**Authors:** Krzysztof Żerdziński, Julita Janiec, Kamil Jóźwik, Paweł Łajczak, Łukasz J. Krzych

**Affiliations:** 1 Students Department “#Intensywna_Po_Godzinach”, Department of Acute Medicine, Faculty of Medical Science in Zabrze, Medical University of Silesia, 41-800 Zabrze, Poland; s88547@365.sum.edu.pl (J.J.); s88169@365.sum.edu.pl (K.J.); s87917@365.sum.edu.pl (P.Ł.); 2 Department of Acute Medicine, Faculty of Medical Science in Zabrze, Medical University of Silesia, 41-800 Zabrze, Poland; lkrzych@sum.edu.pl; 3 Department of Anesthesiology and Intensive Care, Upper-Silesian Medical Center, 40-635 Katowice, Poland

**Keywords:** intensive care, intensive care unit, critical care, artificial intelligence, machine learning, prediction, prognosis, diagnostic accuracy, systematic review, overview of systematic reviews

## Abstract

**Background:** ICU care is time critical and data dense, making it a promising but high-risk setting for AI decision support when tools are weakly validated. ICU AI evidence is heterogeneous, with limited external validation, inconsistent clinically actionable reporting, and scarce real-world impact data, yielding fragmented review conclusions. We mapped five prespecified ICU domains and assessed clinical and implementation maturity to identify key translational gaps. **Methods**: We performed a PRIOR-aligned overview of systematic reviews with prespecified maturity constructs. PubMed, Embase, and Web of Science were searched (title and abstract) on 13 December 2025, supplemented by backward citation searching. Two reviewers screened and extracted data with arbitration, assessed the review-level risk of bias using ROBIS, and synthesized findings without meta-analysis using a SWiM-guided narrative prioritizing AUROC ranges. **Results:** We included 34 systematic reviews (2017–2025) across five ICU domains, dominated by prognostic and early warning applications, mostly in adult populations and commonly using EHR and multimodal inputs. Reporting focused on discrimination, with AUROC ranges roughly 0.54–0.99 for prognostic tasks and 0.64–0.99 for diagnostic tasks, while calibration and clinical utility were rarely addressed and overlap suggested partial dependence. Maturity signals clustered at low-to-intermediate levels, with no evidence for routine, and regulated CDS deployment at the review level. **Conclusions:** Review-level evidence indicates a translational gap between retrospective performance and clinically mature, safely deployable ICU AI, supporting priorities for external validation, prospective impact studies, standardized reporting including calibration, and governance-focused implementation.

## 1. Introduction

Intensive care medicine is defined by high-acuity, time-critical decision making under uncertainty. Clinicians must continuously integrate heterogeneous signals, anticipate rapid deterioration, and balance escalation of organ support against iatrogenic harm, often under staffing and capacity constraints. This combination of physiological complexity, dense longitudinal data, and operational pressure makes the ICU a plausible setting for AI-based decision support and one in which weakly validated tools could plausibly amplify risk [1,2].

AI applications in critical care have expanded across tasks that map to core ICU workflows. Algorithms have been proposed to identify early physiological deterioration, predict adverse outcomes, support the detection of complex syndromes, interpret continuous monitoring streams, and inform treatment decisions in dynamic, feedback-driven care [1,3,4]. At the same time, ICU AI research remains methodologically heterogeneous and often disconnected from implementation realities. Reported performance is frequently difficult to compare across studies because populations, outcomes, time horizons, data modalities, and validation strategies vary substantially. Limited external evaluation, inconsistent reporting of clinically actionable metrics, and sparse evidence on real-world impact and workflow integration further complicate translation from promising prototypes to trustworthy bedside support [1,2,3,4,5,6].

Against this backdrop, the evidence base is increasingly dominated by systematic reviews focused on narrow clinical tasks, single syndromes, specific data modalities, or particular model families. While these reviews are valuable, they can produce fragmented and sometimes discordant conclusions, especially when eligibility criteria, outcome definitions, analytic emphasis, or quality appraisal differ [1,3,4,5]. A reporting guideline for overviews of reviews of healthcare interventions—PRIOR-aligned [7] overview of systematic reviews—is therefore needed to consolidate and critically appraise review-level evidence across the ICU landscape, compare conclusions across domains within a shared clinical frame, and identify recurring limitations that constrain translation into practice.

To preserve clinical relevance, five ICU domains were prespecified to reflect distinct decision problems and implementation pathways. Prognostic and early warning is central because deterioration may be detectable only through subtle temporal patterns, and timely risk stratification can prompt earlier evaluation and treatment. Diagnostic and detection is important because ICU syndromes are often nonspecific, and delayed recognition can lead to preventable harm through late or inappropriate therapy. Monitoring and dynamic assessment is critical because ICU care depends on continuous surveillance, yet alarm fatigue, signal noise, and fragmented displays limit the effective interpretation of high-frequency data. Treatment support and decision support is relevant because critically ill patients show marked heterogeneity in treatment response, and decision support may help personalize strategies while reducing unwarranted variation. Implementation and readiness is essential because technical performance alone does not determine patient benefit, and adoption is constrained by integration, governance, usability, and safety requirements [1,2,3,8,9,10].

This overview uses the concept of clinical maturity to describe how close an AI application is to its credible, clinically meaningful use in intensive care. Clinically mature evidence is expected to be generalizable beyond a single development setting, sufficiently transparent to interpret limitations, and framed to support decision-making rather than statistical optimization alone. This is distinguished from implementation maturity, which reflects whether AI systems have progressed from research outputs to usable, governed tools embedded in real clinical workflows. Consistent with these principles, safe deployment in the ICU is defined here as the use of AI as decision support under conditions that minimize foreseeable patient harm, including credible evaluation beyond development data where available, clear communication of limitations, appropriate human oversight, and governance processes that support monitoring, accountability, and responsible integration into care pathways.

The primary objective of this overview of systematic reviews is to map ICU clinical domains of AI application and characterize clinical maturity across domains using evidence maturity and implementation maturity as prespecified constructs. The secondary objectives are to summarize reported achievements, appraise the methodological quality of the included reviews, and identify and explain discordant conclusions across reviews within domains. The guiding question is as follows: Across prespecified ICU domains, what does the systematic review literature collectively indicate about the maturity and readiness of AI applications for clinically safe and effective adoption, and where are the most consistent gaps that should be prioritized for translational research?

## 2. Materials and Methods

### 2.1. Study Design and Reporting Framework

This study was conducted as an overview of systematic reviews to synthesize and critically appraise evidence on clinical applications of artificial intelligence in intensive care. The unit of analysis was the systematic review level, including umbrella reviews. No primary studies were directly included in the synthesis.

The primary objective was to evaluate the clinical maturity of AI applications across prespecified ICU domains by assessing evidence maturity and implementation maturity, stratified by domain. Evidence maturity reflected the highest level of validation and clinical evaluation reported within included reviews. Implementation maturity captured progression from offline development toward clinical integration.

Methods and reporting followed the Preferred Reporting Items for Overviews of Reviews (PRIOR) statement [7], with explicit attention to overlapping and discordant findings. Because populations, outcomes, AI methods, and validation approaches were heterogeneous, a meta-analysis was not performed. Instead, the results were summarized using a structured narrative synthesis guided by Synthesis Without Meta-analysis (SWiM) [11], with transparent grouping and reporting of performance ranges, direction, and consistency.

The methodology was predefined in a protocol and registered prior to study completion in the International Prospective Register of Systematic Reviews PROSPERO (CRD420251252865). Any protocol deviations were prospectively documented and reported. The full protocol (v1.0), including prespecified domains and maturity definitions, is available in Supplementary File S1.

### 2.2. Eligibility Criteria

Systematic reviews were eligible at the review level, including umbrella reviews, provided they synthesized evidence on AI applications relevant to intensive care and did not report only a single primary study.

Eligible reviews focused on critically ill patients managed in intensive care settings. Adult ICU, PICU, and NICU were prespecified as parallel population categories and coded as equivalent strata during extraction. Reviews were included when an ICU population constituted the majority of the evidence base, or when the cohort clearly reflected an intensive care phenotype. This phenotype was defined as acute or life-threatening illness requiring advanced monitoring and organ support, most commonly respiratory insufficiency, shock, cardiac arrest, or clinical instability requiring close monitoring and frequent laboratory testing. Reviews were required to explicitly address at least one prespecified domain, namely prognostic or early warning, diagnostic or detection, monitoring or dynamic assessment, treatment support or decision support, or implementation or readiness.

For eligibility purposes, a systematic review was defined as a focused investigation addressing a specific question and using explicit, prespecified methods to identify, select, critically appraise, and synthesize all relevant studies, aiming to minimize bias and enhance reproducibility. Non-systematic reviews and non-eligible publication types were excluded, as were records not addressing an ICU clinical task, reviews in which ICU evidence was not extractable or ICU relevance could not be supported, protocols, duplicates or superseded versions, and records without an accessible full-text form.

### 2.3. Information Sources and Search Strategy

PubMed, Embase, and Web of Science were searched using a predefined strategy. The search was performed once on 13 December 2025, without date restrictions. The searches were limited to title and abstract fields, and no additional filters were applied. The full database-specific strategies and execution details are provided in Supplementary Table S1.

Backward citation searching was also performed by screening the reference lists of the included systematic reviews and a small number of key full-text articles assessed at later screening stages to identify additional eligible records.

### 2.4. Study Selection

The records retrieved from database searches were exported to Zotero for automated deduplication, followed by manual checking to remove residual duplicates. The de-duplicated library was then imported into Rayyan for screening.

Two reviewers (K.Ż. and J.J.) independently screened titles and abstracts, followed by a full-text assessment of potentially eligible records. Disagreements were resolved by consensus, with arbitration by a third reviewer (P.Ł.) when a consensus could not be reached.

Near-duplicate systematic reviews were retained during selection, and no review was excluded on the basis of suspected overlap. The overlap between reviews was evaluated post hoc after data extraction as part of the overlap analysis.

At the full-text stage, exclusions were recorded using prespecified categories. The reasons included AI not being applied to an ICU clinical task, being set in the wrong setting or in population where ICU evidence was not extractable, and the full text being unavailable. Reviews without an accessible full text were actively sought through institutional access, academic platforms including ResearchGate, and direct requests for sharing via ResearchGate. If the full text remained unavailable, the record was excluded.

### 2.5. Data Extraction and Data Items

Data were extracted using a structured workbook in Google Sheets. The form was piloted on five randomly selected systematic reviews and refined prior to full extraction. Two reviewers independently extracted key data items, and disagreements were resolved by discussion. The completed screening and extraction workbook, including structured extraction fields and decision log elements, is provided in Supplementary File S2.

A MASTER table was populated to capture bibliographic details, review characteristics, clinical focus, domains, population type, data modalities, AI model categories, validation approaches, comparators, and main performance results. Performance was recorded as key reported ranges. The implementation or translation focus, major methodological limitations, and the data sources used in primary studies were extracted as reported by the reviews. Certainty of evidence was recorded only when explicitly reported by review authors.

The risk of bias was assessed using ROBIS, selected for its fit to the included review designs, and recorded alongside any risk-of-bias assessments reported within the reviews. Evidence maturity and implementation maturity were assigned on a predefined zero to three scale. For items allowing multiple categories, including domains, ICU type, modality, and validation, multiple coding was applied. When data were insufficient or heterogeneous, items were coded as unclear or mixed. No authors were contacted.

### 2.6. Risk of Bias and Quality Assessment

The risk of bias in the included systematic reviews was assessed using ROBIS. The full ROBIS Phase 2 domains were applied, and the optional Phase 1 was not undertaken. Two reviewers assessed each review, and a third reviewer adjudicated unresolved disagreements. The overall review quality was defined as the ROBIS overall risk-of-bias judgment.

The risk of bias in the primary studies was not reassessed. Instead, the approach used by each review to appraise the primary study quality was extracted, and absence of a formal appraisal was treated as a critical limitation when interpreting conclusions.

The reporting bias was assessed descriptively using domain-adapted signals. For prognostic models, these included selective reporting centered on discrimination, missing calibration, and a lack of external or temporal validation. For diagnostic reviews, signals included the incomplete reporting of sensitivity and specificity. For monitoring applications, signals included the omission of false alarm rates or alarm burden metrics.

Certainty statements were recorded when the review authors explicitly reported them. In addition, an overview-level qualitative characterization of interpretability was provided to communicate how strongly findings could be relied upon for inference. This characterization was informed by the ROBIS overall risk of bias, the consistency of findings, and whether the included reviews performed structured a primary study risk-of-bias assessment using established tools. The primary studies’ risk of bias was not reassessed, and where reviews did not appraise the primary studies’ risk of bias, this was treated as a major interpretability constraint and highlighted explicitly in the synthesis.

### 2.7. Handling Overlap and Discordance

Overlap and discordance were handled using a two-step approach. First, a light analysis of overlap was performed as a pragmatic proxy for redundancy within each clinical domain. Overlap-light was defined as the duplicate citation rate within the domain-specific pool of primary studies’ references extracted from included systematic reviews. It was quantified as (input records minus unique records) divided by input records, and classified using prespecified thresholds, with low being below three percent, moderate three to ten percent, or high above ten percent. Because this approach relies on duplicate citation strings, it was interpreted as an indicator of redundancy rather than a direct systematic review of the overlap metric the primary studies.

Second, discordance was assessed after the completion of the domain-specific synthesis. Discordance was defined as meaningful differences in conclusions within a domain and was explored using a prespecified algorithm examining, in sequence, differences in search dates, eligibility criteria, populations or settings, outcomes and performance metrics, and methodological quality. Discordance was considered when interpreting consistency and was used to down-weight narrative certainty.

For reporting, a concise narrative description in the main manuscript is sufficient, while a supplementary table is feasible and preferable. The available domain reports support tabulation of overlap-light counts and the key discordance drivers per domain.

### 2.8. Assessment of Evidence and Implementation Maturity

Evidence maturity was operationalized as a four-level scale reflecting the highest level of model evaluation reported within each clinical domain. Level 0 was assigned when evidence was limited to internal development or internal validation only. Level 1 reflected external or temporal validation. Level 2 reflected prospective evaluation or impact assessment. Level 3 reflected real-world deployment evidence.

Implementation maturity was assessed using a parallel four-level scale. Level 0 corresponded to offline research prototypes. Level 1 captured technical integration within clinical systems. Level 2 reflected supervised clinical decision support use. Level 3 reflected embedded and regulated or operational CDS in routine care.

Maturity ratings were assigned at the domain level and anchored to the level supported by the systematic review with the highest methodological quality, based on full-text data extracted independently by two authors. Because maturity was assigned at the domain level and anchored to the highest-quality review, the domain rating represents the highest maturity signal supported by the most methodologically robust evidence within that domain, rather than the most common maturity level across all reviews. Given the multi-label mapping, the same limited set of reviews can contribute level 2 signals across multiple domains, even when most reviews remain at earlier stages.

Certainty was handled at two levels. Certainty statements were extracted only when explicitly reported by review authors and were recorded in the MASTER table. In addition, an overview-level qualitative characterization of interpretability was provided based on ROBIS overall risk of bias, the consistency of findings across reviews, and the maturity of validation and deployment. The absence of a formal risk-of-bias assessment of primary studies within contributing reviews was treated as a major constraint on interpretation and highlighted explicitly in the narrative synthesis.

### 2.9. Data Synthesis

Data were synthesized using a SWiM-guided [11] structured narrative approach. Synthesis was conducted at the clinical-domain level, with stratified interpretation across population type, data modality, validation features, and evidence and implementation maturity. A meta-analysis was not performed due to substantial clinical and methodological heterogeneity across reviews.

AUROC ranges were prioritized when available. When AUROC was not central, performance measures were retained as presented in the included reviews, using task-appropriate metrics. Heterogeneity was explored narratively by comparing populations and settings, outcome definitions, model types, and validation strategies. Reporting bias was assessed using domain-adapted signals and incorporated into interpretation of the direction of findings.

Interpretability was considered at two levels. Certainty statements provided by review authors were extracted and reported when available. For overview-level interpretation, an overview-level qualitative characterization of interpretability was informed by the ROBIS overall risk-of-bias judgment, the consistency of findings across reviews, and the maturity of validation evidence. The absence of structured risk-of-bias assessments of primary studies within contributing reviews was treated as a major interpretability constraint and was flagged explicitly in the narrative synthesis, rather than being applied as an automatic downgrading rule.

## 3. Results

### 3.1. Study Selection Results

Database searches identified 513 records from PubMed (n = 165), Embase (n = 167), and Web of Science (n = 181). No additional records were identified via other methods. After duplicate removal (n = 275), 238 unique records proceeded to title and abstract screening, and 188 were excluded at this stage. Screening was performed independently by two reviewers, with 21 conflicts resolved by a third reviewer.

**Figure 1:**
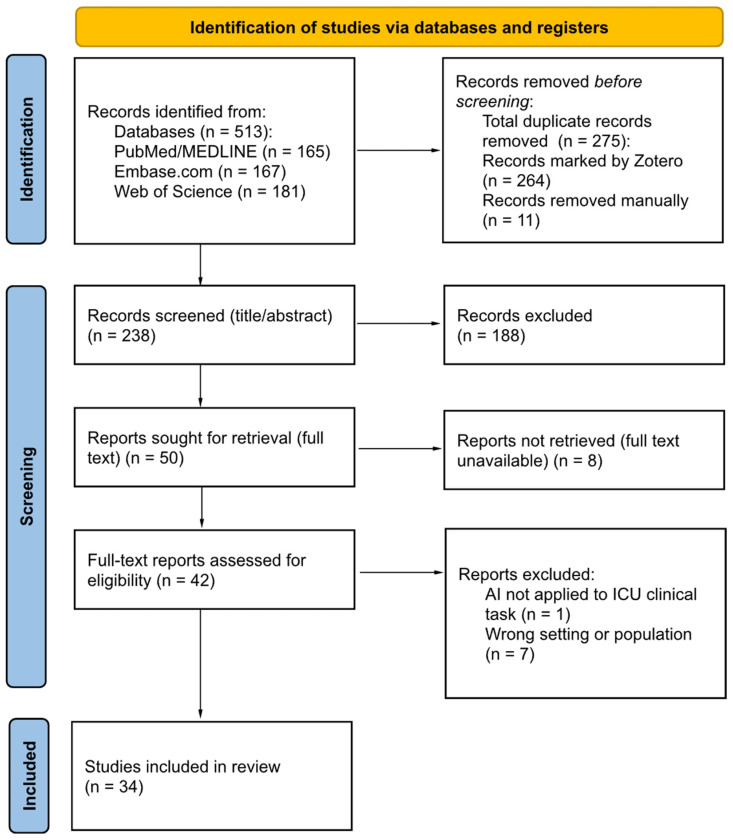
PRISMA 2020 flow diagram [12] adapted for PRIOR-compliant [7] study selection in an overview of systematic reviews.

Fifty reports were sought for full-text retrieval. Eight could not be retrieved, primarily because of unavailability, abstract-only access, or technical PDF access errors. Forty-two full texts were assessed for eligibility, and eight were excluded, most commonly because the ICU setting or population was not extractable (n = 7) or because AI was not applied to an ICU clinical task (n = 1). Overall, 34 systematic reviews were included in the overview. The study selection process is summarized in Figure 1.

### 3.2. Characteristics of Included Reviews

The 34 included systematic reviews were published between 2017 and 2025, with clear clustering in recent years. One review was published in 2017, three in 2019, three in 2021, three in 2022, six in 2023, seven in 2024, and eleven in 2025.

Domain mapping was multi-label, because individual reviews could address more than one ICU task. Prognostic or early warning applications predominated, with 33 reviews mapped to this domain. Diagnostic or detection tasks were addressed in 15 reviews. Implementation or readiness was also covered in 15 reviews. Monitoring or dynamic assessment and treatment or decision support were each covered in eight reviews.

Adult ICU populations were most frequently represented (n = 26). Pediatric (n = 5) and neonatal (n = 7) intensive care settings were also represented across domains, including prognostic, diagnostic, monitoring, treatment support, and implementation readiness. Mixed-population reviews were present (n = 6), while population classification was not unclear for any included review in the evidence map.

Input modalities were also non-mutually exclusive. EHR data (n = 32) and multimodal inputs (n = 19) were most common across domains. Waveforms (n = 18) and imaging (n = 11) were also frequently represented.

The distribution of reviews across clinical domains, stratified by population, data modality, validation, and systematic review quality, is summarized in the evidence map (Table 1), reflecting the multi-label structure of the mapping. A condensed summary of the aims and scope of included systematic reviews is provided in Table 2, while full extracted characteristics are reported in Supplementary Table S2.

### 3.3. Quality Assessment of Included Reviews

**Figure 2:**
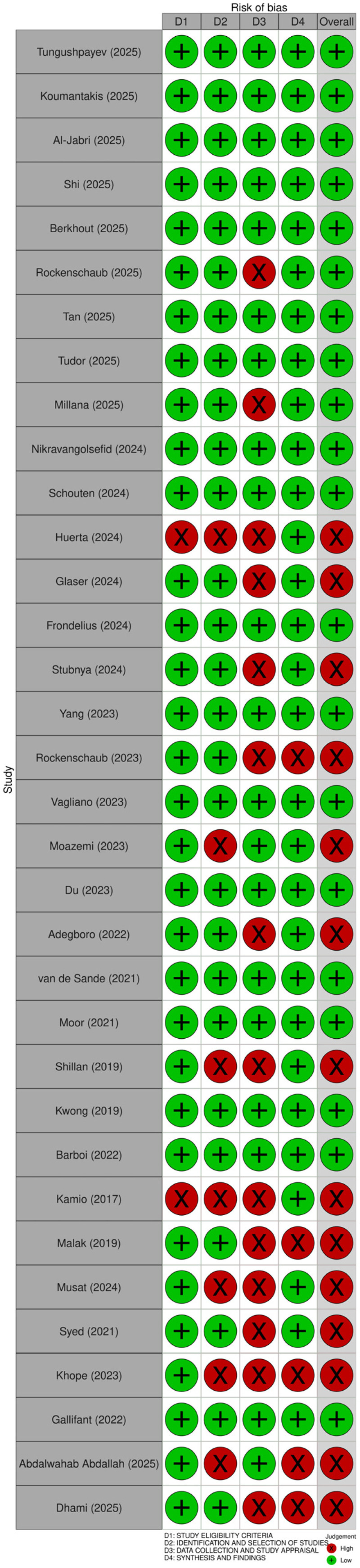
ROBIS risk-of-bias summary for included systematic reviews, overall judgment and key domains [1,2,4,13,14,15,16,17,18,19,20,21,22,23,24,25,26,27,28,29,30,31,32,33,34,35,36,37,38,39,40,41,42,43].

The risk of the bias of the included systematic reviews was assessed using ROBIS. The full Phase 2 domains were applied, and the overall review quality was defined according to the ROBIS overall risk-of-bias judgment, reported as low versus high concerns. Overall, 20 of 34 reviews were judged to have low concerns for bias, while 14 of 34 were judged to have high concerns for bias. A graphical summary of the ROBIS assessment is shown in Figure 2. The review-level ROBIS signaling questions, domain judgments, and overall risk-of-bias ratings are reported in Supplementary Table S3.

High levels of concern were related to the limitations in study identification and selection and to weaknesses in the critical appraisal of the underlying primary evidence. Recurrent issues included the restricted searches relying on a single database or a narrow set of sources, and eligibility decisions shaped by full-text unavailability, including explicit paywall-driven non-retrieval. Another frequent driver was the absence of a formal, validated risk-of-bias or quality assessment for included primary studies, or substitution with ad hoc checklists. These limitations constrained the interpretability of performance estimates and reduced the credibility of review conclusions.

### 3.4. Results by Clinical Domain (SWiM Core)

#### 3.4.1. Clinical Domain: Prognostic and Early Warning Models (SWiM)

This domain comprised 33 systematic reviews. The domain-level distributions of population types, data modalities, and validation approaches are summarized in Table 1.

Discrimination was the dominant performance outcome. Across reviews, AUROC or C-statistic values spanned approximately 0.54 to 0.99 across prognostic and early warning tasks. Reported examples included sepsis prediction AUROC 0.64 to 0.97, AKI prediction 0.55 to 1.00, ICU deterioration or mortality prediction 0.71 to 0.92, and neonatal seizure detection 0.81 to 0.99. Where quantitative pooling was undertaken by review authors, pooled AUROC estimates were typically in the high 0.7 s to low 0.8 s and were accompanied by very high between-study heterogeneity. Sensitivity and specificity were variably reported and often spanned roughly 0.63 to 0.92 and 0.56 to 0.91, respectively, while other metrics such as PPV, NPV, F1, and accuracy were inconsistently synthesized.

Beyond discrimination, calibration and decision-analytic evaluation were rarely addressed, limiting the interpretation of the clinical reliability and the downstream utility.

#### 3.4.2. Clinical Domain: Diagnostic and Detection Models (SWiM)

This domain comprised fifteen systematic reviews, with eight judged as low concern and seven as high concern by ROBIS. The domain-level distributions of population types, data modalities, and validation approaches are summarized in Table 1.

Performance reporting focused on discrimination. Where numeric summaries were provided, AUROC or AUC values were typically in the moderate-to-high range and spanned approximately 0.64 to 0.99 across tasks. Reported examples included pooled AUROC for VAP detection of 0.88 (95% CI 0.82 to 0.94) and early VAP AUROC of 0.84 (95% CI 0.76 to 0.91). Other metrics such as sensitivity, specificity, accuracy, and F1 were variably reported and were rarely synthesized consistently across reviews.

#### 3.4.3. Clinical Domain: Monitoring and Dynamic Assessment Models (SWiM)

This domain included eight systematic reviews published between 2019 and 2025, comprising seven systematic reviews and one umbrella review. The median number of the included primary studies was 41 (range 21 to 262). ROBIS judgments indicated low concern in three reviews and high concern in five. The domain-level distributions of population types, data modalities, and validation approaches are summarized in Table 1.

Performance reporting was heterogeneous and centered on discrimination and accuracy, with examples spanning ROC AUC values around 0.74 to 0.97 and accuracy frequently in the mid-to-high range in task-specific settings. Calibration was repeatedly highlighted as sparse, limiting conclusions about reliability and translation into continuous monitoring workflows.

Reviews clustered into pediatric or neonatal ICU-focused (n = 4), cardiovascular ICU-focused (n = 3), and adult ICU-focused (n = 1), with recurring concerns about heterogeneous outcomes, limited external validation, and inconsistent comparator reporting. Reporting gaps further constrained interpretation, including unclear search date reporting in three of the eight reviews and unclear validation reporting in five of the eight reviews.

#### 3.4.4. Clinical Domain: Treatment Support and Decision Support Models (SWiM)

This domain included eight systematic reviews. ROBIS judgments indicated low concerns in three reviews and high concerns in five. Search reporting was incomplete in three of the eight reviews, and only five of eight provided a directly comparable primary study count, ranging from 21 to 262 (median 32). The evidence maturity was limited (range of one to two; median of two) and implementation maturity remained low overall (range of zero to two; median of 0.5). The domain-level distributions of population types, data modalities, and validation approaches are summarized in Table 1.

Performance reporting was heterogeneous and often not directly comparable across reviews. AUROC or AUC was the most commonly reported metric category (six of eight) followed by sensitivity and specificity (three of eight) and accuracy (three of of eight). Calibration was rarely reported (one of eight). Across the included reviews, the reported examples of discrimination and accuracy spanned broadly across tasks, with AUC or AUROC values often in the approximate 0.70 to 0.99 range in selected applications, while consistent synthesis of calibration and clinical utility remained uncommon.

Treatment and decision support covered multiple workflows, most commonly sepsis-related decision support (five reviews), ventilation-related support (three) extubation (two), triage (two), and vascular access (three). Comparator reporting was frequently unclear, including three of eight reviews flagged as unclear, which limited interpretation of “outperforming” claims.

#### 3.4.5. Clinical Domain: Implementation and Readiness Focused Reviews (SWiM)

This domain comprised 15 systematic reviews. The domain-level distributions of population types, data modalities, and validation approaches are summarized in Table 1, reflecting the heterogeneity in implementation constraints across modalities and settings.

Across the included systematic reviews, performance was typically summarized as AUROC or related discrimination measures, with additional metrics reported variably. Calibration and clinical utility reporting were consistently sparse. The methodological quality varied, with ROBIS low concerns in 10 of 15 and high concerns in 5 of 15, and translation depth was predominantly low (implementation maturity was 0 in 10 of 15).

#### 3.4.6. Cross-Domain Evidence and Implementation Maturity Signals Within Included Reviews

Across all 34 included systematic reviews, evidence maturity signals clustered at the lower to intermediate levels of the prespecified framework. Two reviews (5.9%) were classified as evidence maturity level 0, twenty-three (67.6%) as level 1, and nine (26.5%) as level 2. No review reached an evidence maturity level of 3, indicating an absence of review-level evidence consistent with real-world deployment signals in the underlying primary literature as captured by these reviews.

Implementation maturity was even more concentrated at the earliest stage. Twenty-nine reviews (85.3%) were classified as implementation maturity 0, two (5.9%) as level 1, and three (8.8%) as level 2. No review reached an implementation maturity level of 3. In practical terms, only 5 of 34 reviews (14.7%) contained signals beyond offline evaluation, and all such signals occurred in reviews that also met an evidence maturity level of 2. This cross-domain profile indicates that, even when discrimination performance appears favorable, translation remains constrained by limited prospective impact evidence and sparse movement toward embedded, regulated decision support.

#### 3.4.7. Overlap-Light and Discordance Signals

Overlap-light proxy analysis suggested non-trivial redundancy across domains. The duplicate citation string rates were 18.4% for prognostic and early warning, 12.2% for diagnostic and detection, 6.4% for monitoring and dynamic assessment, 12.2% for treatment support and decision support, and 14.5% for implementation- and readiness-focused reviews.

Discordance was more often explained by differences in scope, target definitions and time windows, population mix, and reporting practices than by opposing directions of performance. In prognostic and early warning, interpretability was the most constrained by sparse calibration and decision-analytic evaluation. In diagnostic and detection, differences in clinical framing were driven by heterogeneity in target definitions and time windows, alongside limited calibration reporting. In monitoring and dynamic assessments, discordance reflected the scope and population mix, with persistent reporting gaps. In treatment support and decision support, discordance was driven primarily by task heterogeneity and unclear comparator reporting. In implementation and readiness, discordance reflected variability in scope, populations, modalities, and evaluation practices. Detailed overlap-light counts and domain-specific discordance drivers are provided in Supplementary File S3.

### 3.5. Evidence and Implementation Maturity Results

To complement performance-focused reporting, clinical maturity was mapped across prespecified ICU domains using two parallel constructs, evidence maturity and implementation maturity. Table 3 provides a domain-level overview of these maturity levels and anchors the mapping to the highest-quality systematic review within each domain.

#### 3.5.1. Maturity Mapping: Prognostic and Early Warning Models

Within the 33 reviews mapped to prognostic and early warning, evidence maturity concentrated in early stages. Two reviews were classified as level 0, twenty-two as level 1, and nine as level 2, with none reaching level 3. This distribution reflects that the literature is dominated by retrospective development and internal validation, with external or temporal validation reported less consistently and prospective or impact evidence appearing only sporadically and typically in small proportions of included studies.

The implementation maturity was more limited. Twenty-eight reviews were classified as level 0, two as level 1, and three as level 2, with none at level 3. Although translation barriers were frequently discussed, explicit indications of technical integration or supervised CDS use were uncommon and generally restricted to a small subset of primary studies within a few readiness-focused reviews.

#### 3.5.2. Maturity Mapping: Diagnostic and Detection Models

In terms of diagnostics and detection, the evidence maturity level remained low to intermediate across 15 reviews. One review was classified as level 0, eight as level 1, and six as level 2, with none at level 3. The pattern is consistent with the predominantly retrospective evidence in which internal validation is the most common evaluation signal, while external or temporal validation and prospective or impact elements are present but limited and not consistently reported across reviews.

The implementation maturity was more constrained. Ten reviews were classified as level 0, two as level 1, and three as level 2, with none at level 3. Higher implementation maturity was concentrated in readiness- or TRL-focused reviews, where a minority of the included systems showed integration or supervised CDS signals, but routine embedded and regulated deployment was not supported at the review level.

#### 3.5.3. Maturity Mapping: Monitoring and Dynamic Assessment Models

Evidence maturity in monitoring and dynamic assessment was predominantly early-stage across eight reviews. One review was classified as level 0, four as level 1, and three as level 2, with none at level 3. This distribution reflects the largely retrospective evidence base with heterogeneous and often incompletely reported validation, and only limited signals consistent with prospective or impact-oriented evaluation.

The implementation maturity was low overall. Six reviews were classified as level 0, one as level 1, and one as level 2, with none at level 3. Where higher implementation maturity was present, it was confined to readiness-oriented syntheses that described isolated integration or supervised CDS signals, without evidence of routine embedded and regulated deployment at the review level.

#### 3.5.4. Treatment Support and Decision Support Models

In terms of treatment support and decision support, the evidence maturity level was predominantly intermediate across eight reviews. Three reviews were classified as level 1 and five as level 2, with none at levels 0 or 3. This profile is consistent with the fact that the literature often reports predictive signal and selected prospective or impact elements, but without review-level evidence that is compatible with real-world deployment.

The implementation maturity remained limited. Four reviews were classified as level 0, one as level 1, and three as level 2, with none at level 3. Higher implementation maturity was concentrated in readiness- or TRL-oriented syntheses that described isolated workflow integration or supervised CDS signals, while routine embedded and regulated decision support was not supported at the review level.

#### 3.5.5. Implementation and Readiness Focused Reviews

Across 15 implementation and readiness reviews, the evidence maturity level concentrated at levels 1 to 2. Eight reviews were classified as level 1 and seven as level 2, with none at level 0 or 3. This reflects synthesis that frequently addresses validation and generalizability signals, but does not provide review-level evidence consistent with routine real-world deployment.

The implementation maturity was higher than in other domains but remained below full embedment. Twelve reviews were classified as level 0, two as level 1, and three as level 2, with none at level 3. Level 1 to 2 signals were largely confined to readiness or TRL-oriented reviews that described limited technical integration and isolated supervised CDS use, while embedded, regulated CDS in routine practice was not supported at the review level.

## 4. Discussion

### 4.1. Principal Findings

Across the 34 included systematic reviews, the overall signal suggested low-to-intermediate evidence maturity with persistently limited readiness for deployment. Most reviews were consistent with evidence maturity levels 1 to 2, with no review-level signal consistent with real-world deployment. The implementation maturity remained largely at level 0, indicating that the reported performance has not translated into embedded, operational CDS at scale.

The largest evidence base addressed prognostic and early warning applications and relied predominantly on EHR-derived features, often within multimodal models. Reporting was dominated by discrimination metrics, with AUROC or C-statistic values spanning roughly 0.54 to 0.99, while calibration and decision-analytic evaluation were seldom addressed, which limits bedside interpretability and escalation planning. Diagnostic and detection tasks were typically multimodal, combining EHR, waveforms, and imaging, and review quality was mixed. Discrimination was generally moderate to high, with AUC values approximately 0.64 to 0.99, including pooled AUROC estimates for ventilator-associated pneumonia detection around 0.88 and early detection around 0.84, but external validation and calibration remained inconsistently reported. Monitoring and dynamic assessment evidence was smaller and highly heterogeneous, often using physiologic-signal-rich inputs and frequent multimodality. Performance reporting again centered on discrimination, with task-specific AUC examples around 0.74 to 0.97, while calibration was repeatedly described as sparse and validation reporting was often unclear. Treatment and decision support reviews covered diverse workflows and outcomes, which limited comparability and synthesis. Discrimination was frequently reported as favorable in selected applications, often within an approximate AUC range of 0.70 to 0.99, yet calibration and consistent comparator reporting were uncommon, and implementation maturity remained low overall. Implementation and readiness-focused reviews explicitly discussed translation barriers, but validation was most often internal and real-world deployment evidence was rare, aligning with a conservative readiness narrative.

### 4.2. Interpretation in Context

The interpretation of the domain-level signals should be weighted by three recurrent determinants. First, reviews’ quality was frequently limited. Only fourteen of thirty-three prognostic and early warning reviews and five of fifteen implementation and readiness reviews were assessed as higher quality, whereas monitoring and treatment support reviews had a larger share of higher-quality reviews—three of eight in each domain. This matters for clinicians and ICU leadership because optimistic performance summaries from lower-quality reviews provide weaker grounds for escalation protocols, staffing, or procurement decisions.

Second, redundancy was non-trivial. Overlap-light signals imply that apparent cross-review consistency may partly reflect dependence on the shared core literature rather than independent replication, which should temper confidence when similar conclusions recur across multiple reviews.

Third, data provenance and evaluation design likely shaped the observed performance patterns. Across ICU AI applications, discrimination can remain favorable even when generalizability is uncertain, because many primary studies are retrospective and may repeatedly draw on public datasets or closely related institutional cohorts. Aggregate AUROC ranges therefore primarily indicate signal detection under study conditions, not that thresholds, calibration, and workflow effects are sufficiently characterized for safe operational use.

These constraints manifest differently across domains. Prognostic and early warning modeling is extensive but heterogeneous in prediction horizons and outcomes. Diagnostic and detection models, often multimodal, show generally favorable discrimination, yet discordance in clinical applicability is expected when case definitions, time windows, and validation practices differ. Monitoring and dynamic assessment models are particularly vulnerable to dataset shift driven by device and practice variation, even when internal performance is strong. Treatment and decision support evidence is the most sensitive to these issues because predictive performance is not equivalent to improved decisions. Implementation- and readiness-focused reviews emphasize reproducibility, explainability, integration burden, and safety and governance barriers, which coherently align with the low implementation maturity observed across the portfolio.

### 4.3. Clinical Maturity and Implications

Clinical maturity mapping indicates that most ICU AI applications remain closer to research prototypes than to operational CDS. Across the full set of 34 systematic reviews, the evidence maturity concentrated at levels 0 to 2, with no review reaching level 3, and implementation maturity remained predominantly at level 0. Only 5 of 34 reviews contained any signals beyond offline evaluation, and these signals were confined to reviews that also met the evidence maturity level of 2. This profile supports conservative implications for clinicians and ICU leadership. Current evidence is generally sufficient to justify further prospective evaluation and tightly governed pilots in selected use cases, but it does not support routine embedded and regulated CDS as a determinant of triage, escalation, or therapy selection.

Accordingly, near-term clinical use should be framed as decision support with explicit human accountability, not as automation. Even where discrimination appears favorable, translation requires model calibration in the target setting, prespecified thresholds linked to actions, and monitoring for drift and unintended consequences. This is particularly salient in ICU workflows, where false reassurance or alarm inflation can directly affect resource allocation and patient harm. The limited prevalence of implementation maturity level 1 to 2 signals suggests that integration and supervised CDS use remain exceptions rather than the norm, and should be treated as pilot-level capabilities that require formal governance rather than informal bedside adoption.

Governance requirements should be considered part of clinical readiness. AI in intensive care relies on highly sensitive longitudinal data and must follow core data protection principles, including lawfulness, transparency, and data minimization under GDPR [44]. The secondary use of ICU data for model development and updating will increasingly intersect with the European Health Data Space framework for health data access and reuse, which is intended to enable research while strengthening control and governance [45,46]. Equity and safety concerns further argue for continuous auditability and the capacity to recalibrate or withdraw models when performance differs across populations or settings. Human-in-the-loop design is therefore not optional. Interfaces should communicate uncertainty and limitations, reduce automation bias, and preserve clinician autonomy.

Regulation aligns with these maturity-based constraints. In the EU, AI-enabled software used for diagnostic or therapeutic decision support intersects with medical device requirements for medical device software, and AI-specific obligations under the AI Act emphasize human oversight and lifecycle monitoring for high-risk systems [47,48]. For ICU leadership, the practical implication is straightforward. Only institutionally governed, transparent decision support systems with defined responsibility, risk management, and post-deployment monitoring are ethically and operationally acceptable. Experimental algorithms should remain in research environments or controlled pilots until they meet these maturity and governance criteria.

The SWOT-based narrative review [49], although not ICU-specific, converges with ICU translation challenges by separating performance potential from deployment reality. It highlights strengths such as improved diagnostic precision, faster processing, predictive modeling, and real-time monitoring. It emphasizes weaknesses that map directly to bedside adoption barriers, including the need for accurate labeled data, high implementation and training costs, dependence on consistently high-quality inputs, and integration challenges with electronic health records. It also frames threats that are particularly salient in high-risk environments like the ICU, namely algorithmic bias affecting vulnerable groups, data privacy and security risks, over-reliance on automated outputs without adequate human oversight, and regulatory hurdles that impede safe implementation and trust.

### 4.4. Gaps and Future Directions

Across domains, the most consequential gaps were the limited transition from retrospective discrimination-focused evaluation to prospective, workflow-integrated assessment, and the persistent absence of reliability and utility evidence required for bedside decisions. Prospective or impact-oriented evaluation was consistently less common than internal or temporal validation, and real-world evaluation was rare, even in domains with large evidence bases. Calibration and decision-analytic evaluation were repeatedly reported as sparse, which constrains translation because high AUROC does not establish trustworthy risk estimates, threshold behavior, or net clinical benefit in ICU escalation pathways. Uneven reporting quality at the systematic review level remains an additional constraint, due to restricted searches, paywall-driven non-retrieval, and the absence of formal or validated quality assessment approaches in some reviews, which collectively reduce the confidence in performance summaries and inflate the uncertainty around generalizability.

In prognostic and early warning models, future work should prioritize the prospective evaluation of clinically aligned prediction targets, horizons, and actionability, rather than incremental optimization of retrospective performance. The current evidence base is large, but prospective or impact evaluation remains less frequent and real-world evidence is exceptional, while calibration and decision-analytic evaluation are rarely addressed. Standardized outcome definitions, the transparent handling of time-varying data and missingness, and the explicit evaluation of alert burden, false positives, and downstream workload effects remain priorities.

For diagnostic and detection tasks, heterogeneity in target definitions and time windows, combined with sparse external validation and limited calibration reporting, continues to drive discordance and limits transferability across units. Priorities include multi-center and temporal external validation in settings that reflect local microbiology, imaging practices, and case mix, alongside evaluation of decision consequences such as unnecessary treatment escalation or delayed recognition when sensitivity and specificity trade-offs shift in practice. Clear reporting of validation strategies remains necessary given the proportion of reviews with unclear validation reporting.

Monitoring and dynamic assessment models face distinct scalability barriers linked to device heterogeneity, signal processing pipelines, and rapid dataset shift, amplified by incomplete validation reporting and repeatedly sparse calibration evidence. Future directions should emphasize real-time evaluation within representative monitoring environments, explicit drift detection and model updating strategies, and the standardized reporting of populations and operating contexts, given the documented reporting gaps in search and validation fields within this domain.

Treatment support and decision support models require a stronger shift from predictive accuracy toward demonstrable improvement in decisions and outcomes. Comparator reporting was frequently unclear and calibration reporting was rare, which limit the interpretation of outperforming claims and undermine the readiness for protocol-level adoption. Future studies should predefine decision points, comparators, and safety endpoints, and then evaluate impacts using prospective designs that capture clinician behavior, resource use, and unintended consequences, rather than relying on retrospective discrimination.

Finally, implementation- and readiness-focused evidence highlights that operationalization remains the central bottleneck. Prospective or impact evaluation is less common and real-world deployment evidence is rare, while translation depth remains predominantly low. Future directions should prioritize interoperable integration, end-user co-design, auditable documentation, and post-deployment monitoring plans, and should start with lower-risk applications that can be supervised and iteratively scaled. Given the non-trivial redundancy observed across domain bibliographies, future evidence syntheses should strengthen overlap assessment at the primary-study level to avoid double counting and to support the clearer prioritization of genuinely independent evidence.

### 4.5. Limitations

This overview inherits the limitations of the underlying systematic reviews and available reporting. Across domains, performance reporting was heterogeneous and frequently centered on discrimination, while calibration and other decision-relevant metrics were inconsistently addressed, which limits inferences about reliability and bedside utility in high-stakes ICU workflows. The methodological quality of the included reviews was mixed, with several domains containing a substantial proportion of reviews with high risks of bias, alongside domain-specific reporting gaps that constrained interpretation, including unclear validation reporting and incomplete reporting of search dates in monitoring-focused evidence. In treatment and decision support, additional constraints arose from incomplete search reporting and frequent uncertainty in comparator reporting, which limits how confidently outperformance claims can be interpreted across heterogeneous workflows.

Several limitations were specific to the overview methods and scope. The risk of bias was not reassessed at the primary study level, and the interpretation relied on how the review authors appraised primary studies, treating absent or ad hoc appraisal as a critical limitation. A meta-analysis was not undertaken because of the substantial clinical and methodological heterogeneity across reviews, so the conclusions rest on a SWiM-guided structured narrative synthesis that cannot provide pooled estimates and is more sensitive to reporting variability. Full-text unavailability also influenced eligibility despite active attempts to retrieve inaccessible reports, which may have introduced selection bias if non-retrieved reviews differed systematically from the included evidence.

Finally, overlap was assessed using an overlap-light proxy based on duplicate citation strings within domain bibliographies. This indicates redundancy but does not quantify true systematic review to primary study overlap and can under- or overestimate dependence across reviews. Under this proxy, redundancy was highest in prognostic and early warning models (18.4% duplicate rate), and remained non-trivial in diagnostic and detection (12.2%), monitoring and dynamic assessment (6.4%), treatment and decision support (12.2%), and implementation and readiness (14.5%). This elevates the risk of double-counting the apparent breadth of evidence, particularly in domains with higher redundancy, and reinforces the need to interpret convergence across reviews as potentially driven by shared primary study cores rather than independent replication.

## 5. Conclusions

This overview of 34 systematic reviews published between 2017 and 2025 mapped AI applications across five prespecified ICU domains and revealed an uneven evidence landscape dominated by prognostic and early warning use cases, largely in adult populations and most often based on EHR and multimodal inputs. Across domains, systematic reviews primarily reported discrimination metrics, most commonly AUROC, whereas calibration, clinical utility, and validation were inconsistently reported and frequently insufficient, limiting interpretability and transportability. Clinical maturity mapping indicated that the field remains concentrated in early stages, with relatively limited external and prospective evaluation and no review-level signal supporting routine, embedded, regulated clinical decision support deployment. The methodological quality was variable, with important concerns in a subset of reviews, and discordant conclusions were largely attributable to differences in scope, populations, task definitions, and reporting practices, alongside non-trivial redundancy within domain bibliographies. Overall, the findings delineate a persistent translational gap and support prioritizing robust external validation, prospective impact evaluation, standardized reporting including calibration, and implementation-focused governance as prerequisites for safe and scalable clinical adoption.

## Figures and Tables

**Table 1:** Condensed characteristics of included systematic reviews. P = Prognostic/Early warning; D = diagnostic/detection; M = monitoring/dynamic assessment; T = treatment/decision support; and I = implementation/readiness.

Author, Year	Aim of Study	Domain(s) (Multilabel)	Population Type	Data Modality	Validation Approach	Implementation/Translation Focus
Tungushpayev (2025) [2]	Explore ML/DL for ICU management and outcomes across diagnosis, prognosis, and treatment.	P, D, M, T, I	Mixed	Multimodal (EHR, Imaging, Waveforms)	Internal, External/Temporal, Prospective/Impact	Yes
Koumantakis (2025) [13]	Systematically review and meta-analyze DL models for ICU readmission prediction and performance.	P, I	Adult ICU	EHR	Internal, External/Temporal, Unclear	Yes
Al-Jabri (2025) [14]	Review ICU delirium ML/DL prediction models, and assess performance, quality, and limitations.	P	Adult ICU	Multimodal (EHR, Waveforms)	Internal, External/Temporal, Prospective/Impact	Yes
Shi (2025) [15]	Evaluate AI-based AKI prediction in ICU, focusing on methods, data use, and applicability.	P	Adult ICU	EHR	Internal, External/Temporal	Yes
Berkhout (2025) [1]	Assess ICU AI operationalization over time, including TRL-based maturity and risk of bias.	I, P, D, T	Adult ICU	Multimodal (EHR, Imaging)	Internal, External/Temporal, Prospective/Impact	Yes
Rockenschaub (2025) [16]	Quantify external validation frequency for ML ICU scores and AUROC change on new hospitals.	P, I	Adult ICU	EHR	External/Temporal, Unclear	Yes
Tan (2025) [17]	Evaluate ML for early ARDS mortality prediction versus conventional scores and limitations.	P	Mixed	EHR	Internal, External/Temporal	Yes
Tudor (2025) [18]	Map NICU AI for outcome and length-of-stay prediction, including benefits and challenges.	P, I	NICU	Multimodal (EHR, Imaging)	Unclear	Yes
Millana (2025) [19]	Review AI in NICUs across prognosis, classification, monitoring, and forecasting, with integration issues.	P, D, M	NICU	Multimodal (Waveforms, Imaging, Other)	Unclear	Yes
Nikravangolsefid (2024) [20]	Synthesize sepsis ICU mortality ML models, including validation, calibration, and comparators.	P	Adult ICU	EHR	Internal, External/Temporal	Unclear
Schouten (2024) [21]	Assess maturity and risk of bias of AI models used during NICU/PICU stay.	P, D, M, T, I	NICU, PICU	Unclear	Internal, External/Temporal, Prospective/Impact, Real-world	Yes
Huerta (2024) [22]	Map CICU AI applications across key clinical workflows and use cases.	P, D, M, T, I	CICU	Multimodal (EHR, Imaging, Waveforms)	Unclear	Yes
Glaser (2024) [23]	Review ML for predicting and detecting new-onset atrial fibrillation in ICU.	P, D	Adult ICU	Multimodal (EHR, Waveforms)	External/Temporal	Yes
Frondelius (2024) [24]	Compare ML VAP prediction performance and assess interpretability, TRL, and risk of bias.	P, D	Adult ICU	Multimodal (EHR, Waveforms)	Internal, External/Temporal	Yes
Stubnya (2024) [25]	Summarize clinical evidence for ML prediction of sepsis-associated AKI in adult ICU sepsis.	P	Adult ICU	EHR	Internal, External/Temporal	Unclear
Yang (2023) [26]	Evaluate ML model performance for predicting sepsis onset.	P	Adult ICU, Other	EHR, Waveforms	Internal, External/Temporal	No
Rockenschaub (2023) [27]	Assess external validation frequency for ML ICU scores and performance in new hospitals.	P	Adult ICU	EHR	Internal, External/Temporal	Yes
Vagliano (2023) [28]	Critically appraise ICU mortality prognostic models using clinical note embeddings.	P	Adult ICU	EHR	Internal, External/Temporal	No
Moazemi (2023) [29]	Review AI for monitoring-focused clinical decision support in cardiovascular ICUs.	P, D, M, T, I	CICU	Multimodal (EHR, Waveforms)	Internal	Yes
Du (2023) [30]	Assess ML prediction of AKI risk among ICU patients.	P	Adult ICU	EHR	Internal, External/Temporal, Prospective/Impact	Yes
Adegboro (2022) [31]	Review neonatal and pediatric ICU AI for outcomes improvement and real-world readiness.	P, D, T, I	NICU, PICU	Multimodal (EHR, Waveforms, Imaging, Other)	Internal, External/Temporal, Prospective/Impact	Yes
van de Sande (2021) [4]	Assess ICU AI maturity, methods, risk of bias, clinical readiness, and trial outcomes.	P, D, I	Adult ICU	Multimodal (EHR, Waveforms, Imaging, Other)	Internal, External/Temporal, Prospective/Impact, Real-world	Yes
Moor (2021) [32]	Systematically review ML for sepsis onset prediction in adult ICU.	I, P, D	Adult ICU	Multimodal (EHR, Waveforms, Imaging, Other)	Internal, External/Temporal, Prospective/Impact, Real-world	Yes
Shillan (2019) [33]	Review ML on routinely collected ICU data by purpose, methods, validation, and accuracy.	P	Mixed	EHR	Internal, External/Temporal	Yes
Kwong (2019) [34]	Assess the effectiveness of ML for weaning in mechanically ventilated ICU patients.	I	Mixed	Multimodal (Waveforms, EHR)	Unclear	Yes
Barboi (2022) [35]	Meta-analyze ML versus severity scores for ICU mortality prediction and provide guidance.	P	Adult ICU	EHR	Internal, External/Temporal	Limited
Kamio (2017) [36]	Review ML for predicting clinical deterioration in critically ill patients, including utility.	P	Adult ICU	Multimodal (Waveforms, EHR)	Unclear	No
Malak (2019) [37]	Review AI techniques for NICU decision support across diagnosis, prognosis, and monitoring.	P, D, M, T, I	NICU	Multimodal (EHR, Waveforms, Imaging)	Unclear	Yes
Musat (2024) [38]	Review ML models for mortality prediction in critically ill sepsis using routine EMR data.	P	Adult ICU	EHR	Internal, External/Temporal, Prospective/Impact	Yes
Syed (2021) [39]	Review ICU ML applications using the MIMIC dataset.	P, M	Adult ICU	Multimodal (EHR, Waveforms, Other)	Internal, Unclear	Yes
Khope (2023) [40]	Review MIMIC-III analytics and propose a predictive framework.	P, D	Adult ICU	EHR	Unclear	Limited
Gallifant (2022) [41]	Synthesize limitations and solutions for AI in mechanical ventilation, including TRIPOD and PROBAST.	P, D, I	Mixed	Multimodal (EHR, Waveforms)	Prospective/Impact, Internal, External/Temporal	Yes
Abdalwahab Abdallah (2025) [42]	Evaluate AI in PICUs for bias risk, adoption barriers, validation gaps, and readiness.	P, D, M, T, I	Mixed	Multimodal (EHR, Waveforms, Imaging, Other)	Unclear	Yes
Dhami (2025) [43]	Evaluate ICU in-hospital mortality AI/ML models versus traditional scoring systems.	P	Adult ICU	Multimodal (EHR, Waveforms, Imaging)	Internal, External/Temporal	Yes

**Table 2:** Evidence map of included systematic reviews across prespecified ICU domains, stratified by population type, data modality, and validation approach.

	Prognostic and Early Warning Models	Diagnostic and Detection Models	Monitoring and Dynamic Assessment Models	Treatment Support and Decision Support Models	Implementation and Readiness Focused Reviews
n SR	33	15	8	8	15
Population (n SR): Adult ICU	24	7	2	2	7
Population (n SR): PICU	5	4	3	4	4
Population (n SR): NICU	7	6	5	5	6
Population (n SR): CICU	3	3	3	3	3
Population (n SR): Other	1	0	0	0	0
Population (n SR): Mixed	5	3	2	2	4
Population (n SR): Unclear	0	0	0	0	0
Modality (n SR): EHR	31	13	6	7	14
Modality (n SR): Waveforms	17	12	7	6	10
Modality (n SR): Imaging	11	9	5	6	9
Modality (n SR): Multimodal	18	13	7	7	12
Modality (n SR): Other	6	5	3	2	4
Validation (n SR): Internal	28	11	6	6	12
Validation (n SR): External/Temporal	26	10	4	5	11
Validation (n SR): Prospective/Impact	10	8	3	5	8
Validation (n SR): Real-world	1	1	1	1	1
Validation (n SR): Unclear	10	5	5	3	7

**Table 3:** Evidence maturity and implementation maturity by clinical domain in ICU AI (levels 0–3), anchored to the highest-quality systematic review within each domain.

	Prognostic and Early Warning Models	Diagnostic and Detection Models	Monitoring and Dynamic Assessment Models	Treatment Support and Decision Support Models	Implementation- and Readiness-Focused Reviews
n SR	33	15	8	8	15
SR quality (n SR): High	14	7	5	5	5
SR quality (n SR): Low	19	8	3	3	10
Clinical maturity	2	2	2	2	2
Implementation maturity	2	2	2	2	2
Validation (n SR): Internal	28	11	6	6	12
Validation (n SR): External/Temporal	26	10	4	5	11
Validation (n SR): Prospective/Impact	10	8	3	5	8
Validation (n SR): Real-world	1	1	1	1	1
Validation (n SR): Unclear	10	5	5	3	7

## Data Availability

Not applicable.

## Supplementary Materials

The following supporting information can be downloaded at: https://www.mdpi.com/article/10.3390/jcm15010185/s1. Table S1: Full search strategies for PubMed, Embase, and Web of Science. Table S2: MASTER table of included systematic reviews with extracted characteristics, validation approach, comparators, main findings, and major methodological limitations. Table S3: ROBIS Phase 2 signaling questions, domain-level judgements, and overall risk-of-bias rating for each included systematic review. File S1: Full Protocol v1.0, artificial intelligence in intensive care, and an overview of systematic reviews and evidence maturity mapping. File S2: Completed screening and extraction workbook, including extraction fields and decision log elements. File S3: Overlap-light assessment and structured discordance report by clinical domain.

## Abbreviations

ICU	Intensive care unit
AI	Artificial intelligence
ML	Machine learning
PRIOR	A reporting guideline for overviews of reviews of healthcare interventions
SWiM	Synthesis without meta-analysis in systematic reviews: reporting guideline
EHR	Electronic health record
NICU	Neonatal intensive care unit
PICU	Pediatric intensive care unit
CICU	Cardiac intensive care unit
AKI	Acute kidney injury
