# Supplementary material for: Artificial Intelligence in Intensive Care: An Overview of Systematic Reviews with Clinical Maturity and Readiness Mapping
Source: J Clin Med. 2025 Dec 26;15(1):185. doi: 10.3390/jcm15010185 (PMC12786610; doi:10.3390/jcm15010185)
Supplement: Supplementary file 1 [file jcm-15-00185-s001.zip › jcm-4041542-supplementary 2/File S3_ Overlap-light (proxy) analysis by domain.docx]

**Overlap-light (proxy) analysis by domain**

**Artificial Intelligence in Intensive Care: An Overview of Systematic Reviews with Clinical Maturity and Readiness Mapping**

Krzysztof Żerdziński, Julita Janiec, Kamil Jóźwik, Paweł Łajczak, Łukasz J. Krzych

Based on deduplicated domain bibliographies (input total vs unique records). Date: 2025-12-16

Definition used here: overlap-light (proxy) reflects redundancy of citation strings within each domain bibliography. It is not a quantification of SR→primary-study overlap unless each primary study is mapped to each systematic review.

Classification rule (pre-specified for this report): Low <5%; Moderate 5–15%; High >15% duplicate rate, where duplicate rate = (input total − unique) / input total.

## Prognostic / Early warning

**Counts.** Input records: 1932. Unique after deduplication: 1577. Removed as duplicates: 355.

**Duplicate rate.** 18.4% (355/1932). Overlap-light level: High.

Interpretation: a high duplicate rate indicates substantial internal redundancy in the domain bibliography. This increases the risk of double-counting the apparent breadth of evidence if SR conclusions are summarized without checking SR-to-primary-study overlap. Downstream synthesis should prioritize higher-quality and more recent SRs and explicitly note potential dependence across SRs.

## Diagnostic / Detection

**Counts.** Input records: 883. Unique after deduplication: 775. Removed as duplicates: 108.

**Duplicate rate.** 12.2% (108/883). Overlap-light level: Moderate.

Interpretation: a high duplicate rate indicates substantial internal redundancy in the domain bibliography. This increases the risk of double-counting the apparent breadth of evidence if SR conclusions are summarized without checking SR-to-primary-study overlap. Downstream synthesis should prioritize higher-quality and more recent SRs and explicitly note potential dependence across SRs.

## Monitoring / Dynamic assessment

**Counts.** Input records: 512. Unique after deduplication: 479. Removed as duplicates: 33.

**Duplicate rate.** 6.4% (33/512). Overlap-light level: Moderate.

Interpretation: a moderate share of repeated records indicates non-trivial internal redundancy. In practice this often reflects shared core references, repeated guideline/methods citations, or multiple SRs drawing on similar foundational work. This should be considered when narratively weighing how much the evidence base is truly independent.

## Treatment / Decision support

**Counts.** Input records: 485. Unique after deduplication: 426. Removed as duplicates: 59.

**Duplicate rate.** 12.2% (59/485). Overlap-light level: Moderate.

Interpretation: a high duplicate rate indicates substantial internal redundancy in the domain bibliography. This increases the risk of double-counting the apparent breadth of evidence if SR conclusions are summarized without checking SR-to-primary-study overlap. Downstream synthesis should prioritize higher-quality and more recent SRs and explicitly note potential dependence across SRs.

## Implementation / Readiness

**Counts.** Input records: 949. Unique after deduplication: 811. Removed as duplicates: 138.

**Duplicate rate.** 14.5% (138/949). Overlap-light level: Moderate.

Interpretation: a high duplicate rate indicates substantial internal redundancy in the domain bibliography. This increases the risk of double-counting the apparent breadth of evidence if SR conclusions are summarized without checking SR-to-primary-study overlap. Downstream synthesis should prioritize higher-quality and more recent SRs and explicitly note potential dependence across SRs.
