# Supplementary material for: Artificial Intelligence in Intensive Care: An Overview of Systematic Reviews with Clinical Maturity and Readiness Mapping
Source: J Clin Med. 2025 Dec 26;15(1):185. doi: 10.3390/jcm15010185 (PMC12786610; doi:10.3390/jcm15010185)
Supplement: Supplementary file 1 [file jcm-15-00185-s001.zip › jcm-4041542-supplementary 2/File S1_ Full Protocol v1.0, Artificial Intelligence in Intensive Care, an overview of systematic reviews and evidence maturity mapping..docx]

**Full Protocol v1.0**

**Artificial intelligence in intensive care:
An overview of systematic reviews and evidence maturity mapping**

*Date: 13 December 2025*

*Prepared to accompany the PROSPERO registration record (template preview v3).*

**Review team:** Krzysztof Żerdziński (guarantor/contact), Julita Janiec, Kamil Jóźwik, Paweł Łajczak, Łukasz Krzych

**Affiliation:** Department of Acute Medicine, Faculty of Medical Science in Zabrze, Medical University of Silesia, Katowice, Poland

# 1. Review overview

This protocol describes the planned methods for an overview of systematic reviews on artificial intelligence (AI) applications in intensive care and critical care medicine. It aligns with the current PROSPERO record and is written to support transparent, reproducible methods while allowing justified refinements based on the characteristics of the included evidence.

## 1.1 Rationale

AI is increasingly studied in intensive care across diverse clinical tasks, data modalities, and model types. Existing systematic reviews vary substantially in scope, populations, validation standards, and reported outcomes, which fragments the evidence base and may limit translation into practice. An overview of systematic reviews is planned to map the evidence, compare domains, and assess evidence and implementation maturity.

## 1.2 Objectives

To conduct an overview of systematic reviews on AI in intensive care and to synthesize evidence transparently using a structured narrative approach guided by SWiM. Planned outputs include evidence mapping, summary of performance and validation characteristics, clinical maturity mapping, assessment of consistency and discordance across reviews, and identification of gaps and research priorities.

## 1.3 Reporting framework

The overview is planned to be reported in line with PRIOR (Preferred Reporting Items for Overviews of Reviews) and to use SWiM (Synthesis Without Meta-analysis) guidance to structure narrative synthesis when quantitative pooling is not appropriate.

# 2. Eligibility criteria

## 2.1 Population

Included: Critically ill patients cared for in an ICU or equivalent critical care setting, as defined in the included systematic reviews. Reviews addressing adult, pediatric, or neonatal ICU populations are eligible. Reviews are eligible if ICU/critical care is the main focus or if ICU patients constitute a clearly relevant target population within the review.

Excluded: Systematic reviews that do not involve ICU/critical care populations or where a critical care context cannot be reasonably established. Reviews focusing exclusively on non-critical inpatient wards, outpatient settings, primary care, rehabilitation, or long-term care will be excluded. Reviews restricted to non-human data or preclinical studies, and broad AI-in-healthcare reviews without a definable ICU/critical care focus will be excluded.

## 2.2 Intervention/exposure

Included: Systematic reviews of AI/ML/DL models applied to ICU clinical tasks. Planned stratification includes prognostic/early warning, diagnostic/detection, monitoring/dynamic applications, treatment/decision support, and implementation/readiness/operationalization, across any data modality.

Excluded: Reviews not focused on AI/ML/DL and reviews where AI is not applied to ICU/critical care clinical tasks.

## 2.3 Comparators

Systematic reviews will be included regardless of whether they report a formal comparator. Where comparators are reported, comparator type (clinicians/usual care, clinical scores/rules, non-AI statistical models, or other AI models) and the context of comparison (e.g., baseline model vs added AI component) will be extracted. Comparators of limited clinical relevance will be interpreted cautiously and will not drive comparative conclusions.

## 2.4 Outcomes

Primary outcomes: (1) model performance as reported in the included systematic reviews, primarily discrimination (e.g., AUROC/C-statistic; and where relevant sensitivity/specificity, accuracy, F1), summarised as ranges and direction of effect by clinical domain; (2) validation and generalisability signals, including whether external, temporal, prospective, or impact validation is reported; and (3) clinical maturity of AI applications, using prespecified evidence maturity (0-3) and implementation maturity (0-3) ratings based on what is reported.

Additional outcomes: calibration or clinical utility reporting; comparators and incremental value; implementation/translation focus; population and setting descriptors (Adult/PICU/NICU; ICU-only vs mixed); data modality (EHR/tabular, waveforms, imaging, multimodal); evidence gaps and priorities for future research; and notes on discordance and overlap across reviews.

## 2.5 Study designs

Included: Systematic reviews (with or without meta-analysis) evaluating or summarising AI/ML/DL applications for clinically relevant tasks in ICU/critical care. Eligible systematic reviews will be included regardless of the designs of the primary studies they contain (randomised or non-randomised).

Excluded: Narrative/scoping reviews, editorials/commentaries/perspectives, protocols without results, conference abstracts without full text, and primary studies.

## 2.6 Language and date limits

There will be no restrictions on publication year. The overview plans to include systematic reviews published in English.

# 3. Information sources and search strategy

## 3.1 Databases

The planned databases are PubMed, Embase.com, and Web of Science. Searches will be run in title and abstract fields. Full Boolean strategies, dates, and any database-specific limits are planned to be reported in an appendix or supplementary material.

## 3.2 Additional identification methods

Backward and forward citation searching may be used to identify additional eligible systematic reviews missed by database searches. No other sources (e.g., trial registers, dissertations/theses, or conference proceedings) are planned for this overview.

## 3.3 Search updates

If the review process is prolonged, an updated search may be conducted and documented to maintain currency.

# 4. Study selection

Search results will be screened at the systematic review level using a two-stage process (title/abstract followed by full-text assessment). Screening will be performed independently by at least two reviewers, with disagreements resolved by discussion and, if needed, a third reviewer. The selection process will be documented using a PRISMA-style flow diagram at the review level and reasons for full-text exclusions will be recorded.

# 5. Data extraction and data items

Data will be extracted independently by at least two reviewers using a piloted extraction form, with discrepancies resolved by consensus. The team does not plan to contact study authors for additional information; missing or unclear items will be recorded as not reported/unclear and addressed narratively as limitations.

## 5.1 Core data items

A master extraction table is planned to capture, at minimum: publication year; clinical focus; population type (Adult/PICU/NICU); ICU setting (ICU-only vs mixed); sample size ranges; data source(s)/datasets used (including whether public datasets were used); modality (EHR/tabular, waveforms, imaging, multimodal); AI model categories; validation approach (internal/external/temporal/prospective/impact, as reported); comparator (if any); main findings; and major methodological limitations.

## 5.2 Additional data items

Additional items planned include: calibration reporting (yes/no/unclear); clinical utility reporting; implementation/translation focus (yes/no plus key themes); overlap risk noted; and quality/risk-of-bias judgement for each included systematic review.

# 6. Quality and risk-of-bias assessment

Methodological quality/risk of bias of included systematic reviews will be assessed independently by at least two reviewers. One established tool (AMSTAR 2 or ROBIS) is planned to be selected after piloting on an initial subset of reviews, based on feasibility and alignment with the characteristics of the included evidence. Any changes from the planned approach will be reported transparently.

# 7. Risk of bias due to missing results

Risk of bias due to missing results (reporting biases) will be assessed qualitatively, based on indications of selective reporting or publication bias within included systematic reviews (e.g., incomplete outcome reporting, reliance on public datasets, and absence of negative or neutral findings). Potential impacts on interpretation will be described narratively without formal statistical testing.

# 8. Certainty/confidence in the body of evidence

Certainty in the body of evidence will be assessed qualitatively. Confidence judgements will consider the overall quality of the included systematic reviews, the strength of validation and clinical maturity signals, and the consistency of findings across reviews within domains. Given expected heterogeneity, no formal grading system is planned; key sources of uncertainty will be explicitly documented.

# 9. Data synthesis

No formal quantitative data synthesis is planned. Evidence will be synthesised using a structured narrative approach guided by SWiM. Findings will be grouped using prespecified clinical domains, with stratification by population group, modality, validation characteristics, and maturity where feasible. Performance will be summarised as reported ranges and direction of effect, and conclusions will be explicitly linked to the underlying systematic reviews.

## 9.1 Evidence mapping

An evidence map is planned to summarise the distribution of systematic reviews across clinical domains and key modifiers (population group, modality, validation level, and maturity).

## 9.2 Discordance and overlap

Where conclusions differ across systematic reviews within the same domain, discordance will be explored using a prespecified framework considering differences in search dates, eligibility criteria, populations/settings, outcomes/metrics, and review quality. Overlap of primary studies across reviews will be assessed in an overlap-light manner, focusing on key primary studies and search periods within domains.

# 10. Amendments

Any protocol amendments will be recorded with dates and rationale and will be reported in the final manuscript. Where relevant, the PROSPERO record will be updated to reflect major changes.

# 11. Ethics and dissemination

Ethics approval is not required because this overview synthesises findings from published systematic reviews and does not involve individual participant data. The team plans to submit the results for peer-reviewed publication and disseminate findings through academic presentations as appropriate.

# 12. Timeline and status

Planned review timeline (as recorded in the PROSPERO draft): start date 4 November 2025; anticipated end date 10 January 2026. Review status is planned/ongoing at the time of protocol preparation.

# 13. Funding and conflicts of interest

The review has no specific/external funding and is supported by non-commercial institutional affiliations of the review team. No additional conflicts of interest have been declared beyond those recorded for individual team members.

# Appendix A. Clinical maturity framework (planned)

Evidence maturity (0-3): 0 = internal development/validation only; 1 = external and/or temporal validation; 2 = prospective evaluation and/or impact assessment; 3 = real-world deployment evidence.

Implementation maturity (0-3): 0 = offline research prototype; 1 = technical integration in clinical systems; 2 = supervised clinical decision support (CDS) use; 3 = embedded and regulated/operational CDS in routine care. Ratings will be assigned based on information reported in each systematic review and described transparently.
