# Supplementary material for: Artificial Intelligence in Intensive Care: An Overview of Systematic Reviews with Clinical Maturity and Readiness Mapping
Source: J Clin Med. 2025 Dec 26;15(1):185. doi: 10.3390/jcm15010185 (PMC12786610; doi:10.3390/jcm15010185)
Supplement: Supplementary file 1 [file jcm-15-00185-s001.zip › jcm-4041542-supplementary 2/File S2_Screening_and_Extraction_Workbook_COMPLETED.docx]

**Screening and Extraction Workbook**

*AI in Intensive Care - Overview of Systematic Reviews*

**Artificial Intelligence in Intensive Care: An Overview of Systematic Reviews with Clinical Maturity and Readiness Mapping**

Krzysztof Żerdziński, Julita Janiec, Kamil Jóźwik, Paweł Łajczak, Łukasz J. Krzych

**Purpose.** Step-by-step template to document searching, deduplication, screening, full-text eligibility, and data extraction for an overview of systematic reviews. Includes all fields needed to populate an SR-level PRISMA-like flow diagram and the Results 'Study selection' text.

**How to use.** Fill sections in order. Use the same reason labels throughout. If you deviate from the protocol, record it in your Decisions log/Amendments file and note the amendment ID in Section 12.

# 1. Document control

Fill once at project start and update only if needed.

| **Field** | **Entry** |
| --- | --- |
| Review title / short name | Artificial intelligence in intensive care: an overview of systematic reviews and evidence maturity mapping |
| Protocol version (e.g., v1.0) | v1.0 |
| PROSPERO ID (if available) | CRD420251252865 |
| Planned databases | PubMed; Embase; Web of Science |
| Reviewers (R1, R2; third reviewer if needed) | K.Ż.; J.J.; P.Ł. |
| Tooling (reference manager; screening tool) | Zotero (dedup); Rayyan (screening) |
| Workbook created / last updated | 13.12.2025 / 16.12.2025 |
| Reporting guideline(s) | PRIOR (overview of reviews); SWiM (synthesis without meta-analysis). Flow diagram: PRIOR preferred or PRISMA-like adapted for SR-level. |
| Overview-level confidence framework | Pragmatic: SR quality + validation strength + consistency + overlap concerns + primary study RoB + reporting bias. |

# 2. Search execution log (per database)

Run the final search strings. Record dates, platform/interface, any filters, and the number of records retrieved.

| **Database** | **Search run date** | **Platform/ interface** | **Time window/filters** | **Records retrieved (n)** | **Export file name** |
| --- | --- | --- | --- | --- | --- |
| PubMed | 13.12.2025 | PubMed (https://pubmed.ncbi.nlm.nih.gov) | None | 165 | AI_ICU_SR_PubMed_2025-12-13 |
| Embase | 13.12.2025 | Embase.com (Elsevier) | None | 167 | AI_ICU_SR_Embase_2025-12-13 |
| Web of Science | 13.12.2025 | Web of Science Core Collection | None | 181 | AI_ICU_SR_WoS2025-12-13 |
| Update search (optional) | Not performed | N/A | N/A | 0 | N/A |

# 3. Export and deduplication

Recommended workflow: import each database export into Zotero, run 'Duplicate Items', merge duplicates, then export a single deduplicated RIS to Rayyan.

| **Deduplication item** | **Entry** |
| --- | --- |
| Reference manager used | Zotero |
| Import completed | 13.12.2025 |
| Potential duplicates reviewed manually (n) | 22 |
| Records removed as duplicates (total n) | 275 (264 via Zotero + 11 manual) |
| Unique records after deduplication (n) | 238 |
| Deduplicated export file name (RIS/EndNote XML) | AIT_clean_for_Rayyan |

# 4. Rayyan setup (before screening)

Create the Rayyan project, import the deduplicated file, set blinding and labels, and invite reviewers.

| **Setup item** | **Entry** |
| --- | --- |
| Rayyan project name | AiT |
| Import date | 13.12.2025 |
| Records imported (n) | 238 |
| Blinding | Yes |
| Conflict resolution method | Consensus and third reviewer if needed - P.Ł. |
| Reason labels created | Yes |
| Overlap / near-duplicate label created | Tick: [X] Yes [ ] No; If yes: label name: near-duplicate SR |
| SR overlap handling rule during screening | Default: include all eligible SRs; exclude only true duplicates/superseded. If near-duplicate SRs exist, prioritize by recency, higher SR quality, and more robust validation, and document the decision. |
| SR-level discrepant data rule (across SRs) | Default: do not pool; report per SR. If SRs report conflicting values for the same primary study, record the reason (search dates/eligibility/population/metrics/quality) and cite both. |
| Comment (e.g., any import issues) | None |

# 5. Title/abstract screening (SR-level)

Two reviewers screen independently. Record dates, numbers excluded, conflicts, and reason counts.

| **Screening item** | **Entry** |
| --- | --- |
| Start date | 14.12.2025 |
| End date | 14.12.2025 |
| Records screened (n) | 238 |
| Records excluded at title/abstract (n) | 188 |
| Records advanced to full-text retrieval (n) | 50 |
| Conflicts (n) and how resolved | 21 resolved with P.Ł. |
| Records flagged as potential near-duplicate SRs (n) | 0 |
| How flagged/handled (brief) | Label in Rayyan + resolve at full-text using 'Duplicate/superseded SR' rule (Section 7) |

# 6. Full-text retrieval log

Retrieve PDFs for all records advanced to full-text. Do not exclude due to license; record attempts and outcome.

| **Record ID** | **First author (year)** | **DOI/URL** | **Retrieval attempt date(s)** | **Status** | **If not retrieved: reason** | **Notes** |
| --- | --- | --- | --- | --- | --- | --- |
| rayyan-394085116 | Tungushpayev (2025) | 10.1016/j.ijmedinf.2025.106081 | 14.12.2025 | RETRIEVED |  |  |
| rayyan-394085118 | Koumantakis (2025) | 10.1186/s13054-025-05642-x | 14.12.2025 | RETRIEVED |  |  |
| rayyan-394085125 | Al-Jabri (2025) | 10.1016/j.ijmedinf.2025.106008 | 14.12.2025 | RETRIEVED |  |  |
| rayyan-394085128 | Sun (2025) | 10.1111/nicc.70206 | 14.12.2025 | NOT RETRIEVED | Not available |  |
| rayyan-394085139 | Shi (2025) | 10.1093/jamiaopen/ooaf065 | 14.12.2025 | RETRIEVED |  |  |
| rayyan-394085142 | Berkhout (2025) | 10.1001/jamanetworkopen.2025.22866 | 14.12.2025 | RETRIEVED |  |  |
| rayyan-394085155 | Hu (2025) | 10.1111/nicc.13306 | 14.12.2025 | NOT RETRIEVED | Not available |  |
| rayyan-394085156 | Dantas (2025) | 10.1016/j.idh.2024.07.003 | 14.12.2025 | RETRIEVED |  |  |
| rayyan-394085158 | Rockenschaub (2025) | 10.1186/s12911-024-02830-7 | 14.12.2025 | RETRIEVED |  |  |
| rayyan-394085160 | Gomutbutra (2025) | 10.1080/21646821.2025.2520094 | 14.12.2025 | RETRIEVED |  |  |
| rayyan-394085166 | Yang (2025) | 10.2196/66615 | 14.12.2025 | RETRIEVED |  |  |
| rayyan-394085167 | Tan (2025) | 10.2196/70537 | 14.12.2025 | RETRIEVED |  |  |
| rayyan-394085170 | Ji (2025) | 10.1177/08850666251372499 | 14.12.2025 | NOT RETRIEVED | Not available |  |
| rayyan-394085171 | Tudor (2025) | 10.2196/63175 | 14.12.2025 | RETRIEVED |  |  |
| rayyan-394085175 | Millana (2025) | 10.1016/j.siny.2025.101690 | 14.12.2025 | RETRIEVED |  |  |
| rayyan-394085177 | Da Cunha Lyrio (2025) | 10.1097/01.ccm.0001104340.70640.be | 14.12.2025 | NOT RETRIEVED | Abstract-only |  |
| rayyan-394085182 | Nikravangolsefid (2024) | 10.1016/j.jcrc.2024.154889 | 14.12.2025 | RETRIEVED |  |  |
| rayyan-394085184 | Zhang (2024) | 10.3233/THC-240087 | 14.12.2025 | RETRIEVED |  |  |
| rayyan-394085185 | Schouten (2024) | 10.1007/s00134-024-07629-8 | 14.12.2025 | RETRIEVED |  |  |
| rayyan-394085188 | Huerta (2024) | 10.1016/j.pcad.2024.06.006 | 14.12.2025 | RETRIEVED |  |  |
| rayyan-394085191 | Glaser (2024) | 10.1007/s00540-024-03316-6 | 14.12.2025 | RETRIEVED |  |  |
| rayyan-394085193 | Er (2024) | 10.1016/j.jcrc.2024.154581 | 14.12.2025 | NOT RETRIEVED | PDF error |  |
| rayyan-394085199 | Frondelius (2024) | 10.1016/j.ejim.2023.11.009 | 14.12.2025 | RETRIEVED |  |  |
| rayyan-394085203 | Otten (2024) | 10.1097/CCM.0000000000006100 | 14.12.2025 | NOT RETRIEVED | Not available |  |
| rayyan-394085204 | Stubnya (2024) | 10.14744/dcybd.2023.3620 | 14.12.2025 | RETRIEVED |  |  |
| rayyan-394085210 | Nikravangolsefid (2024) | 10.1097/01.ccm.0001003932.70674.67 | 14.12.2025 | NOT RETRIEVED | Abstract-only |  |
| rayyan-394085217 | Yang (2023) | 10.1186/s12879-023-08614-0 | 14.12.2025 | RETRIEVED |  |  |
| rayyan-394085220 | Rockenschaub (2023) | 10.1101/2023.10.11.23296733 | 14.12.2025 | RETRIEVED |  |  |
| rayyan-394085222 | Vagliano (2023) | 10.1016/j.jbi.2023.104504 | 14.12.2025 | RETRIEVED |  |  |
| rayyan-394085227 | Lin (2023) | 10.1007/s12630-023-02445-y | 14.12.2025 | NOT RETRIEVED | Abstract-only |  |
| rayyan-394085230 | Moazemi (2023) | 10.3389/fmed.2023.1109411 | 14.12.2025 | RETRIEVED |  |  |
| rayyan-394085232 | Du (2023) | 10.7717/peerj.16405 | 14.12.2025 | RETRIEVED |  |  |
| rayyan-394085240 | Smit (2022) | 10.1101/2022.10.29.22281684 | 14.12.2025 | RETRIEVED |  |  |
| rayyan-394085247 | Adegboro (2022) | 10.1542/hpeds.2021-006094 | 14.12.2025 | RETRIEVED |  |  |
| rayyan-394085255 | van de Sande (2021) | 10.1007/s00134-021-06446-7 | 14.12.2025 | RETRIEVED |  |  |
| rayyan-394085256 | Moor (2021) | 10.3389/fmed.2021.607952 | 14.12.2025 | RETRIEVED |  |  |
| rayyan-394085257 | Chee (2021) | 10.3390/ijerph18094749 | 14.12.2025 | RETRIEVED |  |  |
| rayyan-394085270 | Wulff (2019) | 10.1055/s-0039-1695717 | 14.12.2025 | RETRIEVED |  |  |
| rayyan-394085271 | Shillan (2019) | 10.1186/s13054-019-2564-9 | 14.12.2025 | RETRIEVED |  |  |
| rayyan-394085275 | Kwong (2019) | 10.1007/s42242-018-0030-1 | 14.12.2025 | RETRIEVED |  |  |
| rayyan-394085284 | Lv (2024) | 10.1186/s12879-024-10380-6 | 14.12.2025 | RETRIEVED |  |  |
| rayyan-394085290 | Barboi (2022) | 10.2196/35293 | 14.12.2025 | RETRIEVED |  |  |
| rayyan-394085291 | Kamio (2017) | N/A | 14.12.2025 | RETRIEVED |  |  |
| rayyan-394085309 | Malak (2019) | 10.1007/s10462-018-9635-1 | 14.12.2025 | RETRIEVED |  |  |
| rayyan-394085314 | Musat (2024) | 10.3390/biomedicines12122892 | 14.12.2025 | RETRIEVED |  |  |
| rayyan-394085318 | Syed (2021) | 10.3390/informatics8010016 | 14.12.2025 | RETRIEVED |  |  |
| rayyan-394085330 | Khope (2023) | 10.3390/healthcare11050710 | 14.12.2025 | RETRIEVED |  |  |
| rayyan-394085342 | Gallifant (2022) | 10.1016/j.bja.2021.09.025 | 14.12.2025 | RETRIEVED |  |  |
| rayyan-394085352 | Abdalwahab Abdallah (2025) | 10.7759/cureus.80142 | 14.12.2025 | RETRIEVED |  |  |
| rayyan-394085356 | Dhami (2025) | 10.7759/cureus.90465 | 14.12.2025 | RETRIEVED |  |  |

# 7. Full-text eligibility decisions

Assess full texts against inclusion/exclusion criteria. Record decisions and reasons for exclusion to populate PRISMA.

Suggested reasons (full-text exclusions - PRISMA reasons):

- Not a systematic review or meta-analysis
- AI not applied to ICU clinical task
- Primary study only (no SR-level synthesis)
- Wrong setting or population (ICU not extractable)
- Wrong focus/outcomes for this overview
- Protocol only
- Duplicate/superseded SR
- Full text unavailable
- Other (specify)

| **Record ID** | **First author (year)** | **Title (short)** | **Decision** | **Exclusion reason** | **Reviewer notes** | **Linked PDF stored at** |
| --- | --- | --- | --- | --- | --- | --- |
| rayyan-394085116 | Tungushpayev (2025) | The value of machine and deep learning | INCLUDE |  |  | Tungushpayev (2025) |
| rayyan-394085118 | Koumantakis (2025) | Deep learning models for ICU readmission | INCLUDE |  |  | Koumantakis (2025) |
| rayyan-394085125 | Al-Jabri (2025) | Performance of machine and deep learning | INCLUDE |  |  | Al-Jabri (2025) |
| rayyan-394085128 | Sun (2025) |  | NOT RETRIEVED | Full text unavailable | Not available | Sun (2025) |
| rayyan-394085139 | Shi (2025) | Artificial intelligence models for predicting AKI | INCLUDE |  |  | Shi (2025) |
| rayyan-394085142 | Berkhout (2025) | Operationalization of Artificial Intelligence | INCLUDE |  |  | Berkhout (2025) |
| rayyan-394085155 | Hu (2025) |  | NOT RETRIEVED | Full text unavailable | Not available | Hu (2025) |
| rayyan-394085156 | Dantas (2025) | Prediction of multidrug-resistant bacteria | EXCLUDE | Wrong setting or population |  | Dantas (2025) |
| rayyan-394085158 | Rockenschaub (2025) | External validation of AI-based scoring systems | INCLUDE |  |  | Rockenschaub (2025) |
| rayyan-394085160 | Gomutbutra (2025) | A Systematic Review and Meta-Analysis Evaluating | EXCLUDE | Wrong setting or population |  | Gomutbutra (2025) |
| rayyan-394085166 | Yang (2025) | Predictive Modeling of ARDS | EXCLUDE | Wrong setting or population |  | Yang (2025) |
| rayyan-394085167 | Tan (2025) | Early Prediction of Mortality Risk in ARDS | INCLUDE |  |  | Tan (2025) |
| rayyan-394085170 | Ji (2025) |  | NOT RETRIEVED | Full text unavailable | Not available | Ji (2025) |
| rayyan-394085171 | Tudor (2025) | Opportunities and Challenges of Using Artificial Intelligence | INCLUDE |  |  | Tudor (2025) |
| rayyan-394085175 | Millana (2025) | A systematic review on the use of artificial intelligence | INCLUDE |  |  | Millana (2025) |
| rayyan-394085177 | Da Cunha Lyrio (2025) |  | NOT RETRIEVED | Full text unavailable | Abstract-only | Da Cunha Lyrio (2025) |
| rayyan-394085182 | Nikravangolsefid (2024) | Machine learning for predicting mortality | INCLUDE |  |  | Nikravangolsefid (2024) |
| rayyan-394085184 | Zhang (2024) | Diagnostic performance of machine-learning | EXCLUDE | Wrong setting or population |  | Zhang (2024) |
| rayyan-394085185 | Schouten (2024) | From bytes to bedside | INCLUDE |  |  | Schouten (2024) |
| rayyan-394085188 | Huerta (2024) | The premise, promise, and perils of AI | INCLUDE |  | CICU | Huerta (2024) |
| rayyan-394085191 | Glaser (2024) | Machine learning in the prediction | INCLUDE |  |  | Glaser (2024) |
| rayyan-394085193 | Er (2024) |  | NOT RETRIEVED | Full text unavailable | PDF error | Er (2024) |
| rayyan-394085199 | Frondelius (2024) | Early prediction of ventilator-associated pneumonia | INCLUDE |  |  | Frondelius (2024) |
| rayyan-394085203 | Otten (2024) |  | NOT RETRIEVED | Full text unavailable | Not available | Otten (2024) |
| rayyan-394085204 | Stubnya (2024) | Machine Learning-Based Prediction of AKI | INCLUDE |  |  | Stubnya (2024) |
| rayyan-394085210 | Nikravangolsefid (2024) |  | NOT RETRIEVED | Full text unavailable | Abstract-only | Nikravangolsefid (2024) |
| rayyan-394085217 | Yang (2023) | Predicting sepsis onset in ICU | INCLUDE |  |  | Yang (2023) |
| rayyan-394085220 | Rockenschaub (2023) | Generalisability of AI-based scoring systems in the ICU | INCLUDE |  |  | Rockenschaub (2023) |
| rayyan-394085222 | Vagliano (2023) | Prognostic models of in-hospital mortality | INCLUDE |  |  | Vagliano (2023) |
| rayyan-394085227 | Lin (2023) |  | NOT RETRIEVED | Full text unavailable | Abstract-only | Lin (2023) |
| rayyan-394085230 | Moazemi (2023) | Artificial intelligence for clinical decision support | INCLUDE |  |  | Moazemi (2023) |
| rayyan-394085232 | Du (2023) | Predictive value of machine learning for the risk of AKI | INCLUDE |  |  | Du (2023) |
| rayyan-394085240 | Smit (2022) | Causal inference using observational ICU data | EXCLUDE | AI not applied to ICU clinical task |  | Smit (2022) |
| rayyan-394085247 | Adegboro (2022) | AI to Improve Health Outcomes in the NICU and PICU | INCLUDE |  |  | Adegboro (2022) |
| rayyan-394085255 | van de Sande (2021) | Moving from bytes to bedside | INCLUDE |  |  | van de Sande (2021) |
| rayyan-394085256 | Moor (2021) | Early Prediction of Sepsis in the ICU | INCLUDE |  |  | Moor (2021) |
| rayyan-394085257 | Chee (2021) | Artificial Intelligence Applications for COVID-19 | EXCLUDE | Wrong setting or population |  | Chee (2021) |
| rayyan-394085270 | Wulff (2019) | Clinical Decision-Support Systems for Detection of SIRS | EXCLUDE | Wrong setting or population |  | Wulff (2019) |
| rayyan-394085271 | Shillan (2019) | Use of ML to analyse routinely collected ICU data | INCLUDE |  |  | Shillan (2019) |
| rayyan-394085275 | Kwong (2019) | The efficacy and effectiveness of ML | INCLUDE |  |  | Kwong (2019) |
| rayyan-394085284 | Lv (2024) | ML for the prediction of mortality | EXCLUDE | Wrong setting or population |  | Lv (2024) |
| rayyan-394085290 | Barboi (2022) | Comparison of Severity of Illness Scores and AI | INCLUDE |  |  | Barboi (2022) |
| rayyan-394085291 | Kamio (2017) | Use of Machine-Learning Approaches to Predict | INCLUDE |  |  | Kamio (2017) |
| rayyan-394085309 | Malak (2019) | NIC decision support systems using AI | INCLUDE |  |  | Malak (2019) |
| rayyan-394085314 | Musat (2024) | ML in Sepsis Outcome Prediction for ICU Patients | INCLUDE |  |  | Musat (2024) |
| rayyan-394085318 | Syed (2021) | Application of Machine Learning in ICU | INCLUDE |  |  | Syed (2021) |
| rayyan-394085330 | Khope (2023) | Strategies of Predictive Schemes | INCLUDE |  |  | Khope (2023) |
| rayyan-394085342 | Gallifant (2022) | AI for mechanical ventilation | INCLUDE |  |  | Gallifant (2022) |
| rayyan-394085352 | Abdalwahab Abdallah (2025) | The Role of AI in Pediatric Intensive Care | INCLUDE |  |  | Abdalwahab Abdallah (2025) |
| rayyan-394085356 | Dhami (2025) | The Prognostic Performance of AI and ML | INCLUDE |  |  | Dhami (2025) |

Reason counts (full-text exclusions - required for PRISMA):

| **Reason label** | **Excluded (n)** |
| --- | --- |
| AI not applied to ICU clinical task | 1 |
| Wrong setting or population (ICU not extractable) | 7 |
| Full text unavailable | 0 (handled as 'not retrieved'; see Section 6) |

# 8. Included systematic reviews registry

Create the final list of included SRs. This list anchors extraction, quality appraisal, overlap checks, and synthesis. (***MASTER Table***)

# 9. Data extraction form (copy once per included SR)

Copy this section for each included SR (SR ID). Fill all mandatory fields. Use it as the source for the MASTER extraction table. (***MASTER Table***)

# 10. Quality assessment of included SRs

Pilot the quality tool on 5 SRs, select AMSTAR 2 or ROBIS, then assess all included SRs. (***Risk of bias table***)

| **Item** | **Entry** |
| --- | --- |
| Pilot completed (YYYY-MM-DD) | 15.12.2025 |
| Tool selected (AMSTAR 2 / ROBIS) | ROBIS |
| Pilot SR IDs | Five SRs sampled across domains and publication years (IDs not recorded here). Full ROBIS assessments are provided in Supplementary Table S3. |
| Overall categories reported in main text | Low / Unclear / High |
| Quality assessment completed (YYYY-MM-DD) | 15.12.2025 |
| Quality table stored at (file path/link) | Supplementary Table S3 (ROBIS assessment table). |
| Notes |  |

# 11. PRISMA-like flow diagram numbers (SR-level)

Fill these fields after screening. They map directly to the flow diagram boxes (prefer the PRIOR flow diagram; if using a PRISMA-like diagram, state that it was adapted for SR-level).

| **PRISMA box label** | **Value (n)** | **Source (where recorded)** |
| --- | --- | --- |
| Records identified from PubMed | 165 | Section 2 |
| Records identified from Embase | 167 | Section 2 |
| Records identified from Web of Science | 181 | Section 2 |
| Total records identified (sum) | 513 | Section 2 |
| Records removed as duplicates | 275 | Section 3 |
| Records after duplicates removed | 238 | Section 3 |
| Records screened (title/abstract) | 238 | Section 5 |
| Records excluded (title/abstract) | 188 | Section 5 |
| Reports sought for retrieval (full-text) | 50 | Section 5/6 |
| Reports not retrieved (full text unavailable) | 8 | Section 6 |
| Full-text reports assessed for eligibility | 42 | Section 7 |
| Full-text reports excluded (with reasons) | 8 | Section 7 |
| Systematic reviews included in the overview | 34 | Section 8 |

# 12. Next-step checklist (after inclusion list is final)

[V] Create the MASTER extraction spreadsheet (Excel/CSV) based on Section 9 fields.

[V] Complete extraction for all included SRs (double-check: domains, modality, validation, comparator, main findings, limitations).

[V] Complete SR quality assessment and store the quality table for the supplement.

[V] Conduct overlap assessment within each domain (overlap-light and, if feasible, citation matrix/CCA). Document in Section 13.

[V] Extract and record primary study risk of bias, reporting bias/missing results, and certainty information reported in each SR (Section 9).

[V] Prepare SWiM-guided synthesis outline per domain (AUC ranges; direction; consistency; validation level; maturity) and document discrepant data handling (Section 13).

[V] Create flow diagram (prefer PRIOR flow or clearly state PRISMA-like diagram is adapted for SR-level) using Section 11 numbers.

[V] Complete overview-level confidence in evidence table and data/materials availability log (Sections 14-15).

## Cross-reference amendments

If any step differs from the protocol, enter the amendment in your Decisions log/Amendments file and note the amendment ID here: ________

# 13. Primary study overlap and discrepant data (overview-level)

Purpose. Document overlap of primary studies across included SRs within each domain and record discrepant data (discordance) and how it was handled in synthesis.

When to fill. After Section 8 (included SR list) and after completing extraction in Section 9.

|  |  |  |  |  |  |
| --- | --- | --- | --- | --- | --- |
| Domain / subgroup | SR IDs contributing | Overlap method used | Overlap extent (None/Low/Mod/High/Unclear) | Key overlaps (primary studies/datasets) - optional | Impact on synthesis / notes |
| All domains (overview-level) | See Supplementary File S3 | Overlap-light assessment based on reported primary studies, key datasets, and search windows within each domain. | See Supplementary File S3 | See Supplementary File S3 | Overlap was considered when interpreting consistency and when prioritising higher-quality, more recent SRs. |
|  |  |  |  |  |  |

Discrepant data log (across SRs). Use standard categories: search dates; eligibility criteria; population/setting; outcomes/metrics; model definitions; SR quality; overlap.

| Domain / ICU task | SR IDs involved | Discordance category | What differs (brief) | Handling decision | Rationale (1-2 lines) |
| --- | --- | --- | --- | --- | --- |
| All domains (overview-level) | See Supplementary File S3 | Search dates; eligibility criteria; population/setting; outcomes/metrics; model definitions; SR quality; overlap | Domain scopes and operational definitions varied across SRs, leading to non-comparable performance summaries. | No quantitative pooling. Structured narrative synthesis per SR, with prioritisation of external/prospective evidence and higher-quality SRs. | Heterogeneity and inconsistent reporting precluded meta-analysis. Discordance was documented and used to contextualise conclusions. |

# 14. Bias and confidence summary (overview-level; PRIOR-required)

Purpose. Summarize primary study risk of bias (as reported in SRs), reporting bias/missing results, overlap concerns, and produce an overall confidence judgement per domain/task/outcome.

Rule. Base confidence primarily on SR quality, validation strength (external/prospective > internal), consistency of findings, and presence of major concerns (primary study RoB, reporting bias, and high overlap). Record brief justification. ***MASTER Table***

| Domain / ICU task / outcome | Evidence profile (population, modality) | Evidence base (SRs, quality, validation) | Bias/overlap (RoB, reporting bias, overlap) | Consistency summary | Overall confidence + rationale |
| --- | --- | --- | --- | --- | --- |
| Prognostic / Early warning | Population: Adult 23/33; Mixed 5/33; NICU 3/33; CICU 2/33. Modality: Multimodal 18/33; EHR/tabular 13/33; other/unclear 2/33. | SRs: 33. ROBIS overall: low 19, high 14. Evidence maturity (0–3): 0: 2, 1: 22, 2: 9 (≥2: 9/33). Implementation maturity (0–3): 0: 28, 1: 2, 2: 3 (≥1: 5/33). | Certainty/confidence: unclear 24/33; not assessed 7/33; low 2/33. Overlap and discordance were assessed separately (Supplementary File S3). | Discrimination metrics (primarily AUROC) were commonly reported, but task definitions, populations, and validation strategies varied across SRs, limiting direct comparability. | Low. The evidence base is dominated by internal or retrospective validation, with limited higher-maturity evidence and mixed SR quality. |
| Diagnostic / Detection | Population: Adult 7/15; Mixed 4/15; NICU 2/15; CICU 2/15. Modality: Multimodal 13/15; EHR/tabular 1/15; other/unclear 1/15. | SRs: 15. ROBIS overall: low 8, high 7. Evidence maturity (0–3): 0: 1, 1: 8, 2: 6 (≥2: 6/15). Implementation maturity (0–3): 0: 10, 1: 2, 2: 3 (≥1: 5/15). | Certainty/confidence: unclear 11/15; not assessed 3/15; low 1/15. Overlap and discordance were assessed separately (Supplementary File S3). | Discrimination metrics (primarily AUROC) were commonly reported, but task definitions, populations, and validation strategies varied across SRs, limiting direct comparability. | Low. The evidence base is dominated by internal or retrospective validation, with limited higher-maturity evidence and mixed SR quality. |
| Monitoring / Dynamic assessment | Population: Adult 1/8; Mixed 3/8; NICU 2/8; CICU 2/8. Modality: Multimodal 7/8; EHR/tabular 0/8; other/unclear 1/8. | SRs: 8. ROBIS overall: low 3, high 5. Evidence maturity (0–3): 0: 1, 1: 4, 2: 3 (≥2: 3/8). Implementation maturity (0–3): 0: 6, 1: 1, 2: 1 (≥1: 2/8). | Certainty/confidence: unclear 6/8; not assessed 2/8; low 0/8. Overlap and discordance were assessed separately (Supplementary File S3). | Discrimination metrics (primarily AUROC) were commonly reported, but task definitions, populations, and validation strategies varied across SRs, limiting direct comparability. | Low. The evidence base is dominated by internal or retrospective validation, with limited higher-maturity evidence and mixed SR quality. |
| Treatment / Decision support | Population: Adult 1/8; Mixed 4/8; NICU 1/8; CICU 2/8. Modality: Multimodal 7/8; EHR/tabular 0/8; other/unclear 1/8. | SRs: 8. ROBIS overall: low 3, high 5. Evidence maturity (0–3): 1: 3, 2: 5 (≥2: 5/8). Implementation maturity (0–3): 0: 4, 1: 1, 2: 3 (≥1: 4/8). | Certainty/confidence: unclear 6/8; not assessed 2/8; low 0/8. Overlap and discordance were assessed separately (Supplementary File S3). | Discrimination metrics (primarily AUROC) were commonly reported, but task definitions, populations, and validation strategies varied across SRs, limiting direct comparability. | Low. The evidence base is dominated by internal or retrospective validation, with limited higher-maturity evidence and mixed SR quality. |
| Implementation / Readiness | Population: Adult 7/15; Mixed 4/15; NICU 2/15; CICU 2/15. Modality: Multimodal 12/15; EHR/tabular 2/15; other/unclear 1/15. | SRs: 15. ROBIS overall: low 10, high 5. Evidence maturity (0–3): 1: 8, 2: 7 (≥2: 7/15). Implementation maturity (0–3): 0: 10, 1: 2, 2: 3 (≥1: 5/15). | Certainty/confidence: unclear 11/15; not assessed 4/15; low 0/15. Overlap and discordance were assessed separately (Supplementary File S3). | Discrimination metrics (primarily AUROC) were commonly reported, but task definitions, populations, and validation strategies varied across SRs, limiting direct comparability. | Low. The evidence base is dominated by internal or retrospective validation, with limited higher-maturity evidence and mixed SR quality. |

# 15. Data and materials availability log (PRIOR item 27)

Record where you will share or archive supporting materials (e.g., OSF/Zenodo/institutional repository or journal supplement). If not shared, state the reason.

| Material | Location (repository/supplement) | File name | Public (Yes/No) | Date uploaded | Notes |
| --- | --- | --- | --- | --- | --- |
| Full search strategies (all databases) | Journal supplementary materials (MDPI) | Table S1 (Search strategies). | Yes | At submission | Database-specific strings, dates, and field restrictions. |
| Deduplication log and deduplicated export | Described in Supplementary File S2 (this workbook). | Deduplicated export: AIT_clean_for_Rayyan.ris (available upon request). | No | N/A | The deduplicated RIS file and Zotero merge log were not uploaded as supplementary materials. |
| Screening decisions export (Rayyan) + exclusion reasons (full text) | Described in Supplementary File S2 (this workbook). | Rayyan export of decisions (available upon request). | No | N/A | Title/abstract and full-text decisions were performed in Rayyan; exports can be provided if requested. |
| MASTER extraction table (SR-level) | Journal supplementary materials (MDPI) | Table S2 (MASTER extraction table). | Yes | At submission | Review-level extraction dataset used for synthesis and evidence mapping. |
| SR quality assessment table (AMSTAR 2 / ROBIS) | Journal supplementary materials (MDPI) | Table S3 (ROBIS assessment table). | Yes | At submission | Review-level ROBIS assessments and overall judgements. |
| Overlap and discrepant data logs (Section 13 tables) and any citation matrix/CCA files | Journal supplementary materials (MDPI) | File S3 (Overlap-light and discordance report). | Yes | At submission | Structured discordance and overlap documentation per domain. |
| Any analysis code used to generate evidence maps/figures (if applicable) | Not applicable. | N/A | N/A | N/A | Figures and tables were prepared using spreadsheet software. If any analysis scripts exist, they can be shared upon request. |
