# Supplementary material for: Artificial Intelligence in Intensive Care: An Overview of Systematic Reviews with Clinical Maturity and Readiness Mapping
Source: J Clin Med. 2025 Dec 26;15(1):185. doi: 10.3390/jcm15010185 (PMC12786610; doi:10.3390/jcm15010185)
Supplement: Supplementary file 1 [file jcm-15-00185-s001.zip › jcm-4041542-supplementary 2/Table S1_ Full search strategies for PubMed, Embase, and Web of Science.docx]

**PubMed**

(

ICU[tiab] OR PICU[tiab] OR NICU[tiab]

OR "intensive care"[tiab] OR "intensive care unit"[tiab] OR "intensive care units"[tiab]

OR "critical care"[tiab] OR "critically ill"[tiab]

)

AND

(

"artificial intelligence"[tiab] OR "machine learning"[tiab] OR "deep learning"[tiab] OR "neural network*"[tiab]

)

AND

(

"systematic review"[tiab]

OR "meta-analysis"[tiab] OR "meta analysis"[tiab] OR meta-analy*[tiab]

OR "umbrella review"[tiab] OR "review of reviews"[tiab] OR "overview of systematic reviews"[tiab]

)

**Embase**

(

ICU:ti,ab OR PICU:ti,ab OR NICU:ti,ab

OR 'intensive care':ti,ab OR 'intensive care unit':ti,ab OR 'intensive care units':ti,ab

OR 'critical care':ti,ab OR 'critically ill':ti,ab

)

AND

(

'artificial intelligence':ti,ab OR 'machine learning':ti,ab OR 'deep learning':ti,ab OR 'neural network*':ti,ab

)

AND

(

'systematic review':ti,ab OR 'meta-analysis':ti,ab OR 'meta analysis':ti,ab OR meta-analy*:ti,ab

OR 'umbrella review':ti,ab OR 'review of reviews':ti,ab OR 'overview of systematic reviews':ti,ab

)

**Web of Science Core Collection**

TS=(

ICU OR PICU OR NICU

OR "intensive care" OR "intensive care unit" OR "intensive care units"

OR "critical care" OR "critically ill"

)

AND

TS=(

"artificial intelligence" OR "machine learning" OR "deep learning" OR "neural network*"

)

AND

TS=(

"systematic review" OR "meta-analysis" OR "meta analysis" OR meta-analy*

OR "umbrella review" OR "review of reviews" OR "overview of systematic reviews"

)
